# PRYNT: a tool for prioritization of disease candidates from proteomics data using a combination of shortest-path and random walk algorithms

**DOI:** 10.1038/s41598-021-85135-3

**Published:** 2021-03-11

**Authors:** Franck Boizard, Bénédicte Buffin-Meyer, Julien Aligon, Olivier Teste, Joost P. Schanstra, Julie Klein

**Affiliations:** 1 Institut National de la Santé et de la Recherche Médicale (INSERM), U1297, Institute of Cardiovascular and Metabolic Disease, 31432 Toulouse, France; 2 Université Toulouse III Paul-Sabatier, 31330 Toulouse, France; 3 Université de Toulouse, UT1, IRIT, (CNRS/UMR 5505), Toulouse, France; 4 Université de Toulouse, UT2J, IRIT, (CNRS/UMR 5505), Toulouse, France

**Keywords:** Computational biology and bioinformatics, Systems biology, Biomarkers, Molecular medicine, Kidney diseases

## Abstract

The urinary proteome is a promising pool of biomarkers of kidney disease. However, the protein changes observed in urine only partially reflect the deregulated mechanisms within kidney tissue. In order to improve on the mechanistic insight based on the urinary protein changes, we developed a new prioritization strategy called PRYNT (PRioritization bY protein NeTwork) that employs a combination of two closeness-based algorithms, shortest-path and random walk, and a contextualized protein–protein interaction (PPI) network, mainly based on clique consolidation of STRING network. To assess the performance of our approach, we evaluated both precision and specificity of PRYNT in prioritizing kidney disease candidates. Using four urinary proteome datasets, PRYNT prioritization performed better than other prioritization methods and tools available in the literature. Moreover, PRYNT performed to a similar, but complementary, extent compared to the upstream regulator analysis from the commercial Ingenuity Pathway Analysis software. In conclusion, PRYNT appears to be a valuable freely accessible tool to predict key proteins indirectly from urinary proteome data. In the future, PRYNT approach could be applied to other biofluids, molecular traits and diseases. The source code is freely available on GitHub at: https://github.com/Boizard/PRYNT and has been integrated as an interactive web apps to improved accessibility (https://github.com/Boizard/PRYNT/tree/master/AppPRYNT).

## Introduction

Kidney diseases can be defined as any chronic or acute disorder that affects renal structure and function^1^. In their most severe form, they are associated with a variety of complications, such as anemia, mineral and bone disorder or cardiovascular disease, leading to overall increased mortality^2^. Causes of renal failure are highly variable and sometimes unknown^3^. Some kidney diseases are monogenic, resulting from modifications in a single gene. Others are more complex and can result from a multifactorial combination of genetic, environmental and additional modifiers such as age, diabetes, smoking or hypertension. The use of high-resolution analytical omics technologies have resulted in major advances in the elucidation of diverse molecular pathophysiological mechanisms associated with kidney disease. While genomics is frequently used to unravel specific mutations in the genome that can increase the risk of developing certain diseases, disease activity is best captured by transcriptome or proteome analysis, as these traits are closer to the phenotype^4^. Moreover, whilst urine has been known for a very long time as a very informative and non-invasive source of potential candidates in the context of kidney disease^5–9^, the molecular changes observed in urine partially reflect the deregulated mechanisms within kidney tissue. Urinary proteins predominately originate (~ 70%) from kidney and urinary tract by mechanisms of secretion and cellular shedding^10–12^. The remaining challenge associated with such analysis is that these techniques require time-consuming validation experiments to try precisely pinpointing the most probable disease candidate from a list of hundreds of potential candidates. Most of these studies considered urinary proteins showing most prominent changes, either based on fold change or p-value, as new promising disease-related candidates. However, not all renal proteins can be found in urine and not all urinary proteins originate from the kidney. Hence, ranking disease proteins solely based on observed urinary changes might limit the complex view of the disease and insight in its pathophysiology.

To help decipher the picture of the deregulated molecular networks and prioritize disease candidates, computational methods and tools have been proposed^13^. Some approaches prioritize candidates based on their similarity to the list of disease-modified genes^14^. These methods use databases (e.g. OMIM), ontologies (e.g. Gene Ontology) or text-mining from literature to assess similarity of sequence (e.g. POCUS^15^), functional annotation (e.g. PANDA^16^, Endeavour^17^, ToppGene^18^) or locus proximity (e.g. OPEN^19^, PhenoRank^20^). Other approaches use biological networks in order to prioritize candidates (e.g. MaxLink^21^, ToppNet^18^). One of the network-based software most commonly used by biologists in order to interpret high-throughput expression data is Ingenuity Pathway Analysis (IPA)^22^. This suite is based on a PPI network containing millions of structured, manually curated experimental observations. In IPA, the “Upstream Regulator Analysis” (URA) algorithm prioritizes disease candidates using in-house causal network approach to elucidate upstream biological causes that can explain the observed molecular changes^23,24^. One of the main limitations hampering the use of IPA is that the software is proprietary and therefore its use cannot be broadly generalized to the biology community. Many other computational prioritization methods already exist^13^. Some are looking for candidates that directly interact with known disease genes, following the principle of “guilt-by-association”^14,25^. Other, such as shortest-path^26^ or random walk^27^ algorithms, further consider the closeness between candidates and known disease genes in a network considering both direct and indirect relationships. Previous studies have shown that closeness-based approaches outperformed direct neighbour-based methods and that combining closeness-based approaches further improved disease candidate prioritization^14,28^. However, most of these strategies have been used to identify disease candidates at the transcriptome level and not at the proteome level. Moreover, to date, none have been tested in the context of biological fluids.

In order to move from this *status quo*, we developed an approach, named PRYNT (PRioritization bY protein NeTwork) that could help expand and fill the gaps of the molecular view, and predict the significance of proteins that were undetectable in the urine. PRYNT is based on the integration of Search Tool for the Retrieval of Interacting (STRING, version 10.5)^29^ PPI network and a combination of shortest-path and random walk, two closeness-based algorithms as it has been previously shown in the literature that this method outperformed other computational methods^14,26–28^. We used PRYNT in the context of two prototypic human kidney diseases: autosomal dominant polycystic kidney disease (ADPKD)^5,9^ and ureteropelvic junction obstruction (UPJ)^6,7^. ADPKD is a well-characterized monogenic kidney disease induced by a mutation of the PKD1 or PKD2 gene. UPJ is a congenital kidney disease resulting from a complex multifactorial combination of genetic and environmental factors. In order to assess the performance of our approach, we first evaluated the precision of PRYNT in prioritizing ADPKD and UPJ disease candidates and compared it with other methods from recent literature. We also performed an in-depth comparison of the results obtained with PRYNT to two main reference prioritization methods currently used by biologists: prioritization based on experimental results and prioritization based on IPA’s URA algorithm.

## Results

### Contextualization of PRYNT PPI network

**Figure 1:**
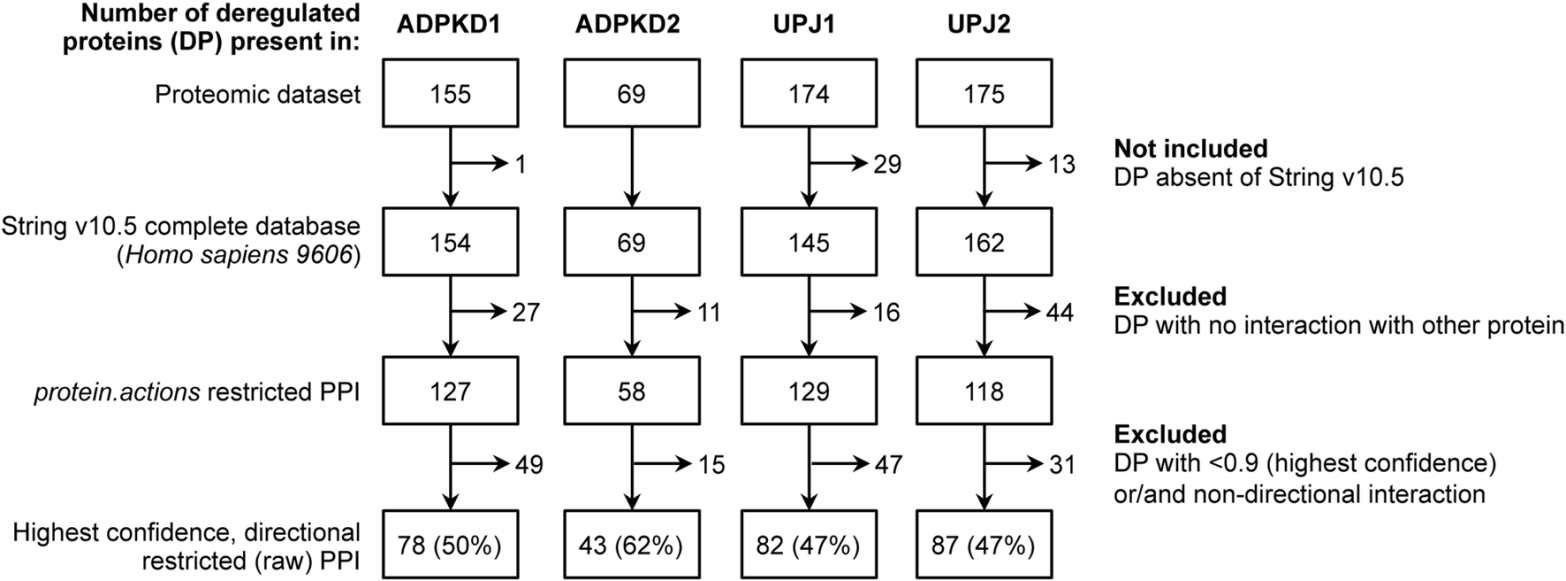
Number of deregulated urinary proteins from proteomic datasets present in the raw PPI network. Part of the deregulated proteins (DP) present in the proteomics datasets could not be included as they were absent in String v10.5 database (Homo sapiens). Moreover, a number of DP was excluded as they did not share any interaction with other proteins (absent from *protein.actions* PPI) or did not have a directional interaction with highest confidence (> = 0.9). PPI: protein–protein interaction network; DP: deregulated protein.

**Figure 2:**
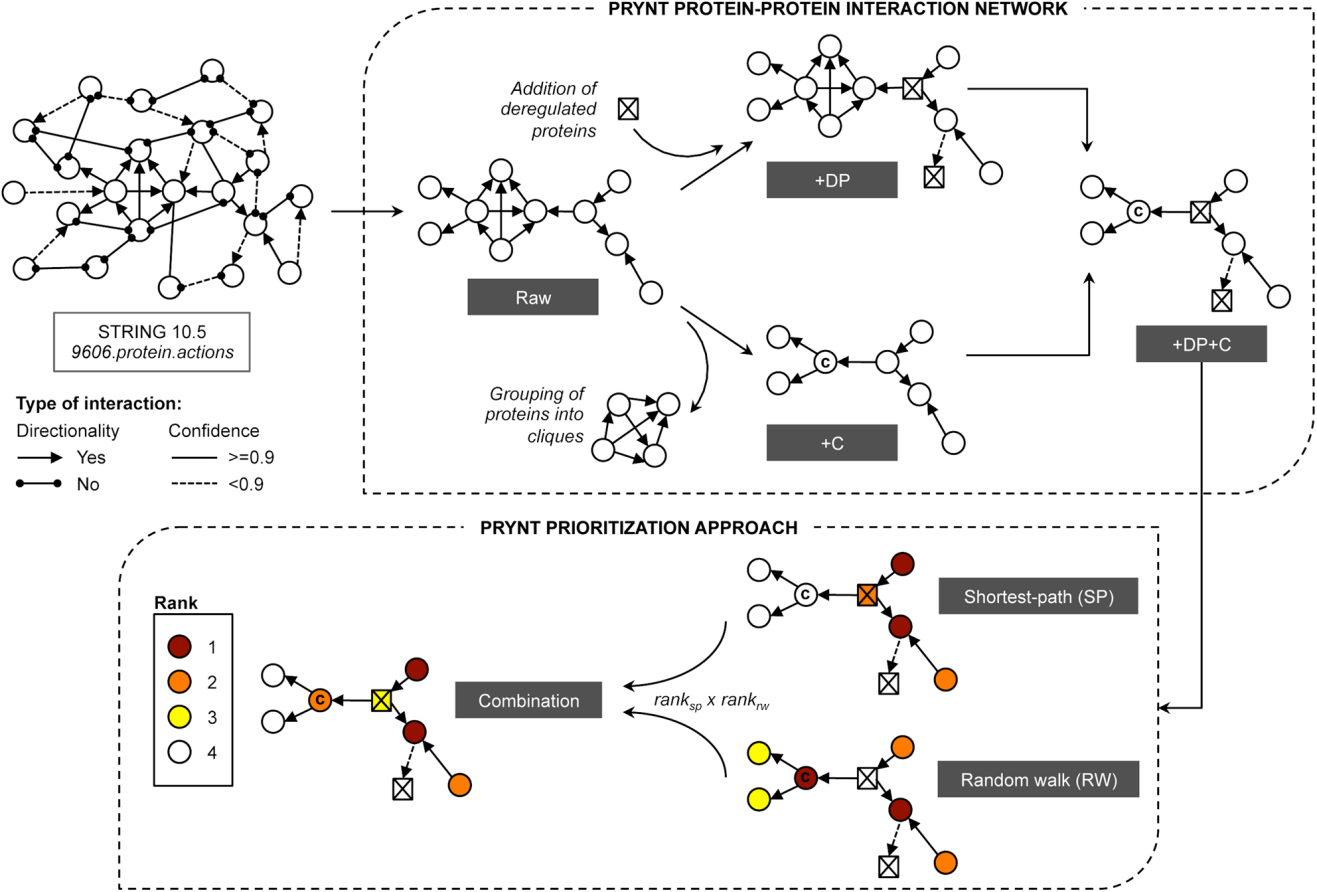
Description of PRYNT algorithm. PRYNT PPI network was based on STRING 10.5 *protein.actions* restricted to Homo sapiens (*9606.protein.actions*), and only directional interaction with confidence >  = 0.9 were selected. The raw PPI network (Raw) was further contextualized by adding the deregulated proteins (+DP) regardless of their confidence level and by grouping the proteins within cliques (+C). PRYNT prioritization approach was based on the combination of shortest-path (SP) and random walk (RW) algorithms and was achieved by multiplying the rank of the protein with the shortest-path ranking strategy (ranksp), and the rank of the protein with the random walk strategy (rankrw). PPI: protein–protein interaction network; DP: deregulated proteins; C: clique.

**Figure 3:**
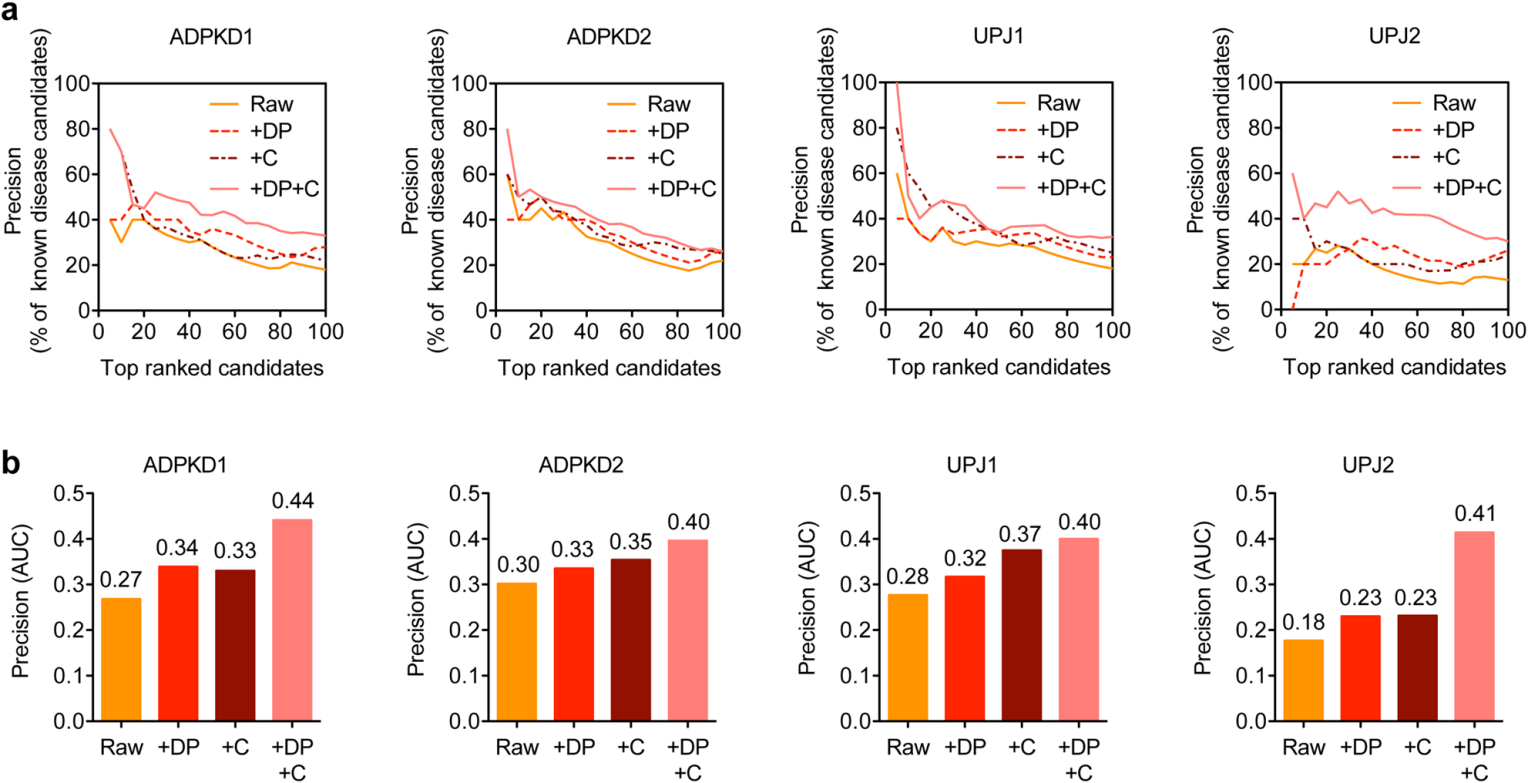
Performance of PRYNT depending on PPI network contextualization. (**a**) The precision was calculated based on the percentage of reference ADPKD or UPJ disease candidates that were prioritized in the top 100 candidates ranked by PRYNT in the four datasets using either the raw PPI network (Raw) or the PPI networks contextualized by the addition of deregulated urinary proteins regardless of their confidence level (+DP), by the management of clique sub-graphs (+C) or by the combination of both (+DP +C). (**b**) The corresponding area under the precision curve (AUC) was calculated in the four datasets. Graphs were designed using GraphPad Prism version 5.0 for Mac, GraphPad Software, San Diego, California USA, http://www.graphpad.com. DP: deregulated protein; C: clique.

In order to test PRYNT approach, four urinary proteome datasets were used: two associated with ADPKD (ADPKD1 and ADPKD2) and two associated with UPJ (UPJ1 and UPJ2) (Table 1 and Supplementary Tables S1–S4). We constructed a PPI network based on STRING database. Approximately 50–60% of the deregulated urinary proteins from ADPKD and UPJ proteomic datasets were present in the raw PPI network (Fig. 1). This rather low percentage could be explained either because part of the deregulated proteins were absent from STRING v10.5 database altogether, or because they did not match the STRING settings that were selected i.e. sharing a *protein.actions* interactions with other proteins in the network, directional interaction and interaction reaching the highest confidence level (Fig. 1). Moreover, 56% (3569 proteins) of the 6391 proteins present in the network were grouped in 265 cliques, which are sets of proteins that all interact with each other and often share similar biological functions. In order to assess the impact of the missing biological input and of the presence of clique sub-graphs in the network, we modified the raw PPI network into three additional contextualized PPI networks (Fig. 2). The first contextualization consisted in generating a PPI network where the deregulated urinary proteins were added regardless of their confidence level (Fig. 2, +DP). The second contextualization consisted in generating a PPI network where cliques were taken into account (Fig. 2, +C). The last network combined both contextualization strategies (Fig. 2, +DP +C). We applied the prioritization strategy combining shortest path and random walk on the four different PPI networks on the four proteomics datasets (Fig. 2). We compared the ranked lists to a list of 500 reference disease candidates of ADPKD for ADPKD1 and ADPKD2, and of UPJ for UPJ1 and UPJ2. The precision was plotted (Fig. 3a) and the areas under the precision curves (AUC) were compared (Fig. 3b). Compared to the raw PPI, the use of the contextualized PPI + DP and PPI + C networks slightly increased the AUC of the precision. However, in the four datasets, the combined PPI + DP + C showed much better performance in terms of prioritizing disease candidates. Based on these results, we generated a contextualized PRYNT PPI network combining both the addition of the deregulated proteins and the management of the cliques (Fig. 2).

**Table 1:** Dataset description.

	Reference	Type of kidney disease	Controls	Cases	Deregulated proteins
ADPKD1	Bakun et al.^5^	Monogenic	30	30	155
ADPKD2	Rauniyar et al.^9^	Monogenic	18	14	69
UPJ1	Lacroix et al.^7^	Complex	10	8	174
UPJ2	Chen et al.^6^	Complex	23	23	175

### Precision of PRYNT compared to other approaches

**Figure 4:**
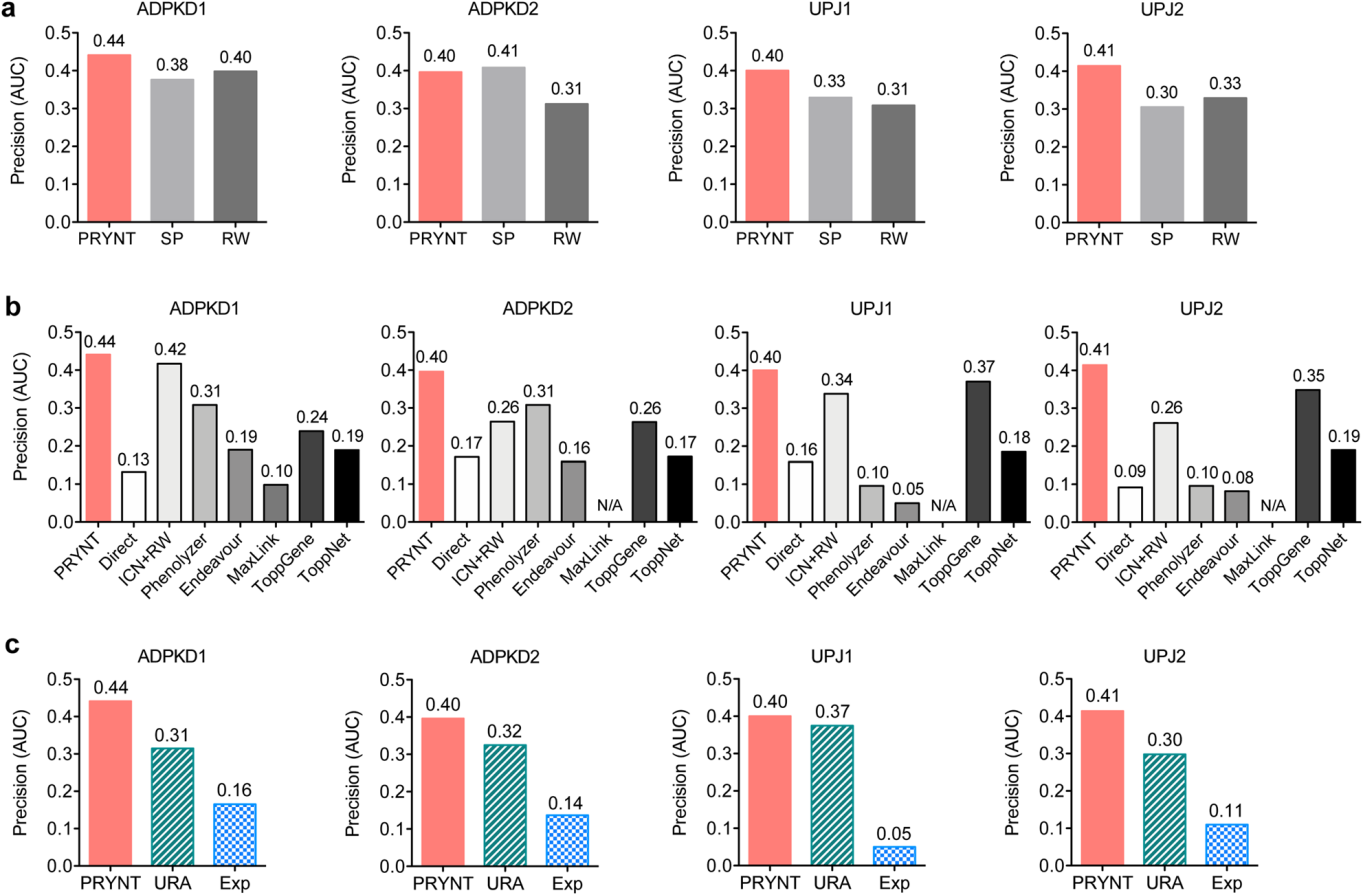
Performance of PRYNT compared to other approaches. PRYNT performance was compared to prioritization using shortest-path or random walk algorithms alone (**a**), to prioritization by other common, state of the art prioritization strategies (**b**), or to prioritization by reference approaches (**c**). The precision was calculated based on the percentage of reference ADPKD or UPJ disease candidates that were prioritized in the top 100 candidates ranked by the different strategies in the four datasets. The corresponding area under the precision curve (AUC) was then calculated. Graphs were designed using GraphPad Prism version 5.0 for Mac, GraphPad Software, San Diego, California USA, http://www.graphpad.com. SP: shortest-path; RW: random walk; D: direct; ICN + RW: interconnectedness combined with random walk; Exp: experimental; URA: upstream regulator analysis.

**Figure 5:**
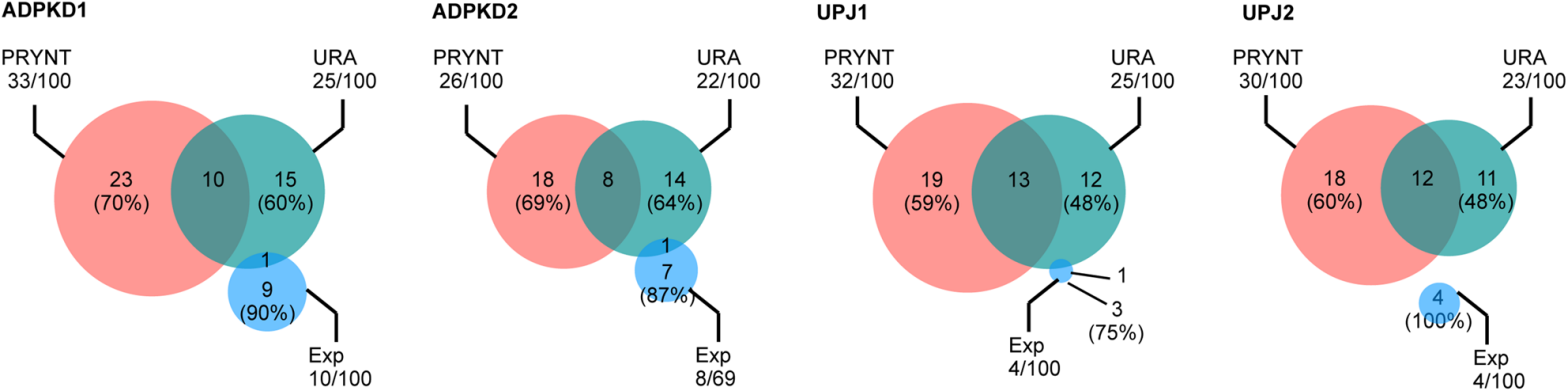
Overlap of reference disease candidates prioritized in the top 100 by PRYNT, URA or Exp. Prioritization by PRYNT, URA or from the experimental urinary proteomic candidates (Exp) was applied and reference ADPKD and UPJ disease candidates ranked in the top 100 were compared in the four datasets. Exp: experimental; URA: upstream regulator analysis.

We first compared PRYNT performance to shortest-path (SP) and random walk (RW) prioritization methods (Fig. 4a and Supplementary Figure S1). Shortest-path between a disease candidate and a differentially abundant urinary protein is defined by the distance between any protein in the network and the differentially abundant proteins, taking into account the direction of interactions. Random walk with restarts simulates a random walker starting on differentially abundant urinary proteins and moving to their immediate neighbors’ randomly at each step. Each protein in the graph is prioritized by the probability of the random walker reaching it. Overall, PRYNT approach, combining both algorithms, showed better performance compared to the two strategies taken separately. Next, we compared PRYNT to seven additional state-of-the-art prioritization algorithms and tools (Fig. 4b and Supplementary Figure S2): direct ranking (Direct), interconnectedness combined with random walk (ICN + RW), Phenolyzer^30^, Endeavour^17^, MaxLink^21^, ToppGene^18^ and ToppNet^18^. Direct ranking and interconnectedness combined with random walk were applied to String raw PPI network. Direct ranking was performed by applying out-degree centrality as described in the study of Oti et al.^25^. Disease candidates were prioritized based on the number of directly interacting differentially abundant urinary proteins. The interconnectedness-based approach combined with random walk was implemented following the study of Hsu et al.^28^. Phenolyzer, Endeavour and ToppGene are similarity-based prioritization approaches, extracting knowledge from diverse databases such as OMIM, Disease Ontology, or Gene Ontology. ToppNet and MaxLink are network-based prioritization approaches, using k-step markov and neighbor-based algorithms respectively. Overall, PRYNT showed better precision compared with these methods (Fig. 4b and Supplementary Figure S2). In particular, the number of candidates predicted by MaxLink was < 100 so we could not assess the AUC for the precision in the top 100 predicted candidates in ADPKD2, UPJ1 and UPJ2. PRYNT performance was then compared to two reference approaches commonly used by biologists (Fig. 4c and Supplementary Figure S3): URA from IPA (URA), and prioritization based on experimental results (Exp). Except for Exp, all tested approaches so far mine a network to find and rank new disease candidates that are linked to the deregulated proteins, without being in the initial set of deregulated proteins. In Exp however, the deregulated proteins are the disease candidates and their prioritization is based on a p-value ranking, the most significant proteins being the highest ranked candidates. In the four datasets, PRYNT showed higher performance to prioritize reference disease candidates compared to URA and Exp, with better precision and superior AUC (Fig. 4c and Supplementary Figure S3). We next analyzed the overlap of reference disease candidates ranked in the top 100 by PRYNT, URA and Exp in the four datasets (Fig. 5). We observed that only a minority of reference disease candidates prioritized by PRYNT and URA were commonly prioritized by both approaches (59–70% uniquely prioritized by PRYNT and 48–64% uniquely prioritized by URA). For Exp, not only the number of prioritized reference disease candidates was very low, but it also showed very poor overlap with URA and no overlap with PRYNT.

### Specificity of PRYNT compared to reference approaches

**Figure 6:**
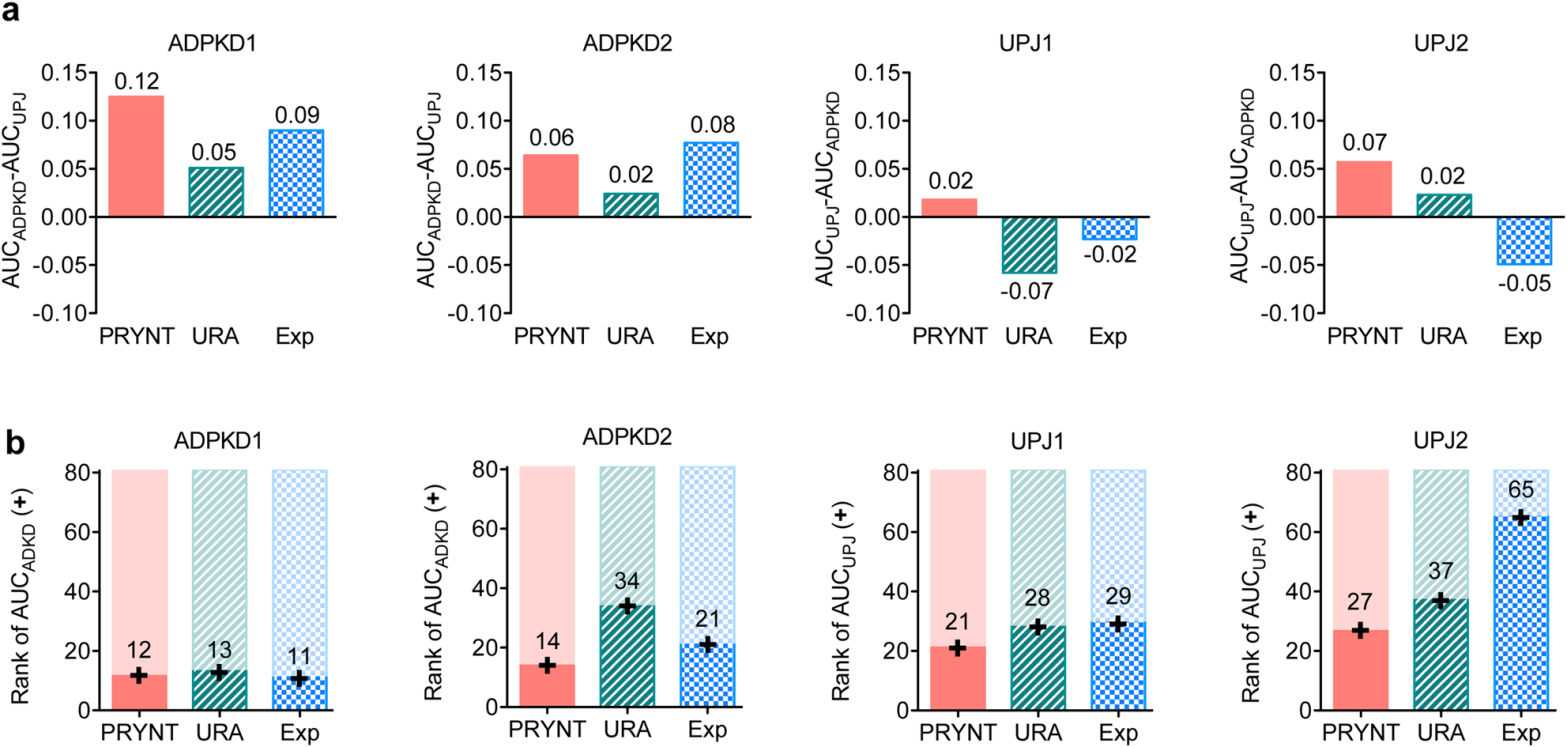
Specificity of PRYNT compared to reference approaches. (**a**) Cross-specificity of the prioritization strategies was assessed for the four datasets by calculating the difference between the AUC of the precision curve for specific disease candidates (AUC_ADPKD_ for ADPKD datasets and AUC_UPJ_ for UPJ datasets) and the AUC of non-specific disease candidates (AUC_UPJ_ for ADPKD datasets and AUC_ADPKD_ for UPJ datasets). (**b**) Overall specificity of the prioritization strategies was assessed for the four datasets by assessing the rank of the AUC of the precision curve for specific reference disease candidates (AUC_ADPKD_ for ADPKD datasets and AUC_UPJ_ for UPJ datasets) compared to 80 additional AUCs of reference candidates from non-specific diseases, including 40 diseases associated to urogenital tract and 40 diseases from other origin. Graphs were designed using GraphPad Prism version 5.0 for Mac, GraphPad Software, San Diego, California USA, http://www.graphpad.com. Exp: experimental, URA: upstream regulator analysis.

We next assessed how the different prioritization strategies ranked candidates that were specific to the disease under study. First, we studied cross-specificity by analyzing whether prioritization in ADPKD datasets was better for specific ADPKD reference disease candidates compared to non-specific UPJ reference disease candidates, and conversely for UPJ datasets (Fig. 6a). For ADPKD1 and ADPKD2, all prioritization strategies showed similar cross-specificity, with the AUC for specific ADPKD candidates (AUC_ADPKD_) being superior to the AUC for non-specific UPJ candidates (AUC_UPJ_). However, for UPJ1 and UPJ2, only PRYNT displayed adequate cross-specificity in both datasets. We next compared overall specificity of the approaches by comparing the AUC of the specific disease to the AUC of 80 non-specific diseases (list in Supplementary Table S5) (Fig. 6b). For APDKD datasets, overall specificity was similar for all strategies in ADPKD1 with the AUC of the specific disease (AUC_ADPKD_) being in the top 15 out of 80 non-specific diseases. In ADPKD2, PRYNT showed better performance compared to URA and Exp (rank of specific AUC_ADPKD_ of 14/81, 34/81 and 21/81 for PRYNT, URA and Exp respectively). For UPJ datasets, overall specificity was lower compared to ADPKD datasets and in both datasets, PRYNT prioritization showed best specificity, with a rank of specific AUC_UPJ_ of 21/81 and 27/81 for UPJ1 and UPJ2 respectively. In UPJ2, Exp showed the lowest specificity with the specific AUC_UPJ_ being ranked 65/81.

### Pathway annotation

**Figure 7:**
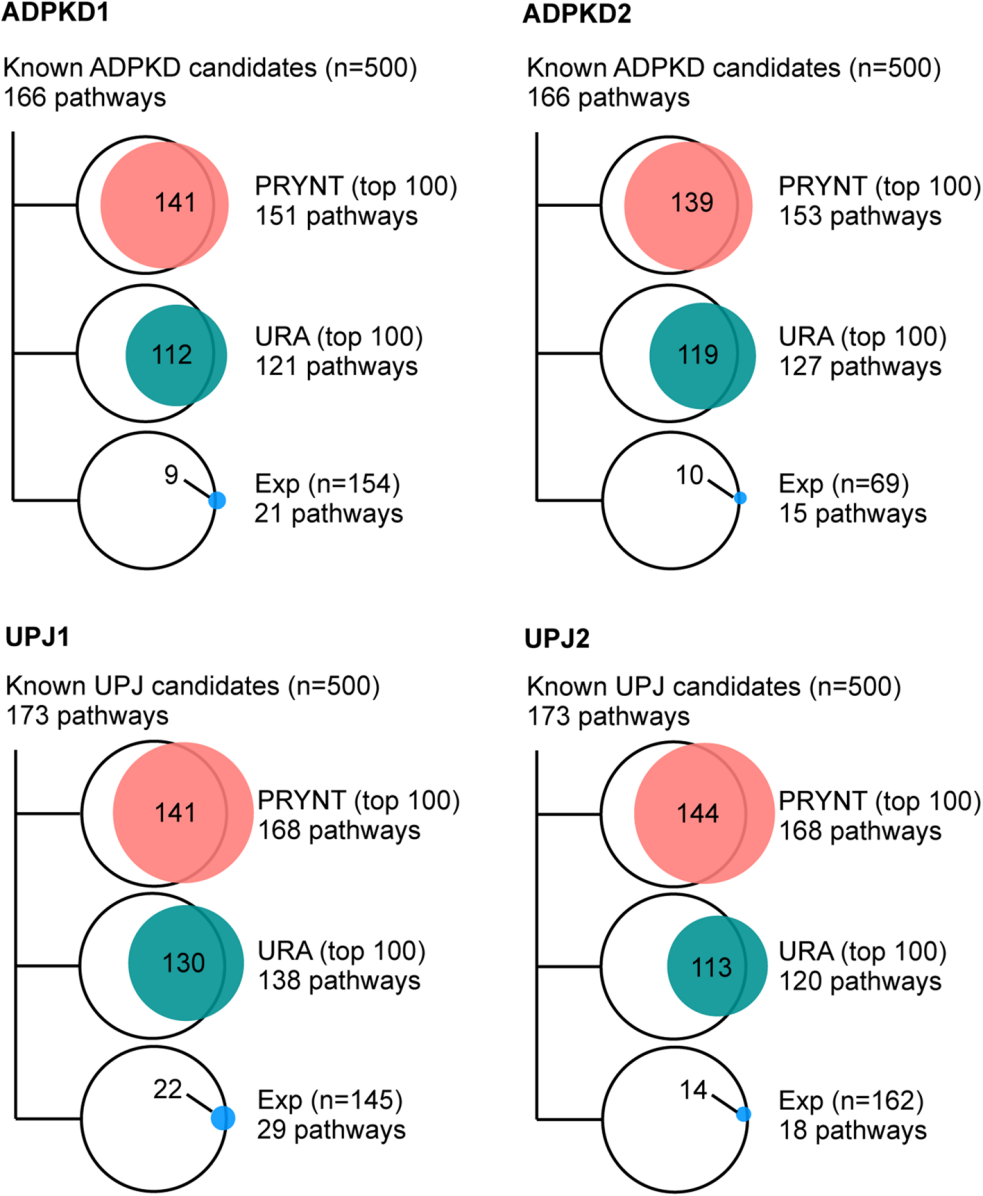
Pathway annotation. KEGG pathway enrichment analysis was applied to the 500 reference ADPKD and UPJ disease candidates, and compared to the pathways enriched from top 100 ranked candidates by PRYNT or URA or from the experimental urinary proteomic candidates (Exp) in the four datasets.

We used KEGG pathway enrichment analysis^31^ to assess the biological relevance of the disease candidates prioritized by PRYNT (Fig. 7). For ADPKD, the 500 reference disease candidates were associated to 166 pathways. Approximately 85% of these pathways were also enriched with the top 100 ranked candidates prioritized by PRYNT (141/166 and 139/166 for ADPKD1 and ADPKD2 respectively) whereas enrichment was 67–72% for URA top 100 (112/166 and 119/166 for ADPKD1 and ADPKD2 respectively) and dropped to approximately 5% for Exp (9/166 and 10/166 for ADPKD1 and ADPKD2 respectively). Similarly for UPJ, PRYNT results showed higher number of enriched pathways and more overlapping pathways associated to the reference UPJ candidates compared to URA and Exp.

### Links of proteins with pathology of interest

Next we assessed the involvement of the top 10 protein candidates in the disease of interest by a systematic search of the scientific literature (Supplementary Tables S6 and S7). For the top ranked ADPKD proteins, all but two were previously linked to ADPKD, confirming the potential of PRYNT in ranking disease candidates (Supplementary Table S6). The two proteins (F2 and HSPA8) not previously linked to ADPKD thus constitute potential candidates for future experiments. All but three of the top 10 proteins were previously linked to UPJ (Supplementary Table S7).

## Discussion

In this study we developed and assessed the performance of PRYNT, a new network-based approach using urinary proteomic profiles to prioritize disease candidates in the context of kidney disease. While many tools and methods are available to predict disease candidates, we developed PRYNT to tackle the specificity of our research question. Indeed, most of these methods, such as Phenolyzer, Endeavour, MaxLink, ToppGene and ToppNet, have been developed on genomic data, seeking for new disease genes and showed that they were less suitable than PRYNT to predict new disease candidates from proteomic data. Combining both shortest-path and random walk, showed better results than using them alone as previously shown by Hsu et al*.*^28^ but also better results than using direct ranking. This latter result proves that closeness-based algorithms are more efficient to mine the PPI network in the context of research on biological fluids, because they are able to select key proteins of kidney disease in the network even though the links between excreted proteins in the urine and modified gene expression at the tissue level (*i.e.* in the kidney) are not necessarily straightforward. Another specificity of PRYNT has been to work on improving the PPI network. To build PRYNT PPI network, we chose to work with STRING database, as it is a well-known, recognized comprehensive database of PPI based on experimental evidence as well as interactions predicted by comparative genomics and text mining. To limit the risk of false prediction, we decided to only select PPI with highest confidence. Two major drawbacks that we identified in this network and using such settings were that a lot of the input information was missing and that the network was massively structured into clique sub-graphs. Instead of using the raw PPI network, we hence decided to contextualize then network by adding the deregulated proteins from the input data, regardless of their confidence, and by grouping the cliques. Cliques are important structures in PPI networks^32,33^. Taking cliques into account by grouping the proteins allowed simplifying the network and helping find the most important disease candidates. As a result, our specific PRYNT contextualized network showed better performance compared to the raw STRING network. We also compared PRYNT to IPA’s URA and to ranking based on experimental results, two references approaches commonly used by biologists. We showed that PRYNT displayed higher precision, higher specificity, and was very informative in terms of pathways and predicted disease candidates.

Whilst PRYNT appears as a valuable method, PRYNT and URA still appear to be complementary as the reference disease candidates short-listed in the top 100 showed rather poor overlap. This confirms that although urine could be a very promising source of biomarkers of kidney disease, complementary methods are still required to allow a closer look into pathophysiology. The consideration of a "tissue-specific" PPI network is an interesting complementary approach for the identification of specific renal mechanisms. This approach has already been used for specialized kidney structures, such as glomeruli^34^ or kidney cells such as podocytes^35^. Such “whole kidney” network is not available yet but it would be a great alternative to current, more global, PRYNT PPI network. With the emergence of multi-omics approaches and the increased importance of additional molecular traits such as miRNA or metabolites in biological research^36–38^, another perspective for PRYNT would be to build an heterogeneous network including several molecular layers (miRNA, mRNA, metabolites, proteins, genes), study them simultaneously, and therefore obtain a more integrated picture of pathologies^39–41^. In the future, PRYNT could evolve towards such application, as the random walk algorithm has already been reported to be efficient in this type of application^42–44^. Another interesting point is that, although a plethora of computational prioritization methods are freely available, the approach most commonly used by biologists still remains the commercial IPA suite. One explanation could be that from a practical point of view, biologists need user-friendly interfaces with little to none required additional programming skills. In this context, we developed a beta version, R-based, web application of PRYNT that can be find in https://github.com/Boizard/PRYNT/tree/master/AppPRYNT. One perspective of this work will be to improve the user experience of PRYNT web application to increase its applicability to the biologists.

In conclusion, the use of the PRYNT could be of great benefit to identify new key proteins associated to renal diseases from urinary proteomic datasets obtained non-invasively. Such approach, that could be applied to any other form of biological fluid and generalized to any other disease, will help fill the gaps and generate the missing links necessary to better understand the deregulated molecular networks, identify new potential biomarkers or develop alternative therapeutic strategies.

## Material and methods

### Urinary proteomic datasets

Study by Bakun et al.^5^ (ADPKD1) analyzed urine protein composition from 30 ADPKD patients and 30 healthy volunteers identifying 155 differentially abundant proteins (Supplementary Table S1). Study by Rauniyar et al.^9^ (ADPKD2) compared 14 urine samples from ADPKD patients to 18 normal controls and identified 69 significantly deregulated proteins (Supplementary Table S2). Lacroix et al.^7^ (UPJ1) explored the urinary proteome of newborns with (n = 8) or without (n = 10) UPJ and discovered 174 differentially abundant proteins (Supplementary Table S3). Chen et al.^6^ (UPJ2) analyzed the proteome of urine from 23 infants with UPJ and 23 controls and identified 175 proteins with different urinary abundance between the two groups (Supplementary Table S4).

### PRYNT algorithm

PRYNT algorithm was developed using R^45^ . PRYNT description can be found in pseudo-code in Supplementary Data S1.

#### Protein–protein interaction network

In the present study, we used STRING *10.5 protein.actions* restricted to Homo sapiens (*9606.protein.actions*), which compiles physical interactions such as reaction, binding, catalysis, inhibition and activation (Fig. 2). Each interaction has a confidence score between 0 and 1 according to the number and the type of source that was used to describe the interaction. Only interactions with the highest confidence level (score greater than 0.9) were selected for PRYNT (Fig. 2). Moreover, directionality of the interaction could be applicable or not to its physical action. Only directional interactions were considered for PRYNT analysis as ranking strategies use directionality (Fig. 2). After removing duplicates and self-linked interactions, we obtained 353643 interactions between 6391 proteins. The raw PPI network was contextualized by adding the deregulated urinary proteins regardless of their confidence level and by removing cliques (Fig. 2).

#### Clique calculation

Applied to a PPI network, a clique is defined as a group of proteins that all interact with each other. Interactions in those cliques in the PRYNT network were considering as undirected, as there is no published method using directionality in cliques. We took into account the maximal cliques of the network, using the R igraph package^46^. In the raw PPI network, 3569 proteins of the 6391 were included in 265 cliques, each clique containing on average 13.5 proteins. We grouped proteins that were part of cliques and selected for each clique the protein candidate with best ranking following prioritization. This led to a PPI network containing 21,051 interactions between 3109 nodes (proteins or cliques).

#### Prioritization approach

Prioritization was based on the combination of two closeness-based approaches, namely shortest-path^26^ and random walk^27^ algorithms. The shortest-path score (SP) of a protein x was calculated as the reciprocal of the sum of the length of the shortest-path between x and the deregulated proteins (y) in the network as specified in Eq. (1).

1 $$SP=\frac{1}{{\sum }_{y}d\left(x,y\right)}$$

The symbol d(x,y) is the minimum number of interaction from x to y. Disease candidates are ranked from higher to lower SP (rank_sp_) (Fig. 2).

The random walk score (RW) corresponds to the probability of a protein to be reached by the walker at the next step t + 1 and can be formally described as follow in Eq. (2).

2 $$RW=\left(1-r\right){AP}_{t}+{rP}_{0}$$

A is the column-normalized adjacency matrix; r the restart probability (set to 0.7 as the default parameters); P_0_ the initial probability of the random walk, i.e. the inverse of the number of deregulated protein for a deregulated protein and 0 for other proteins in the network; and P_t_ the probability after the t-th round of the step. Prioritization based on random walk was calculated using the R package RandomWalkRestartMH^43^. Disease candidates are ranked from higher to lower RW (rank_rw_) (Fig. 2).

For each disease candidate, a combined score (CS) was calculated as defined by Eq. (3).

3 $$CS={rank}_{sp}.{rank}_{rw}$$

Rank_sp_ is the rank of the protein in the shortest-path ranking strategy, and rank_rw_ in the random walk strategy (Fig. 2).

### Additional prioritization methods

#### Additional prioritization algorithms

Direct and interconnectedness combined with random walk (ICN + RW) algorithms have been applied on the raw SRING PPI network. The direct method prioritizes candidates based on whether they directly interact with deregulated urinary proteins^25^. For ICN + RW, the closeness between proteins in the network is quantified by considering not only direct interaction but also the number of connectors between genes^28^.

#### Independent prioritization tools

Recent review from Zolotareva et al*.*^13^ described 14 up-to-date and available gene prioritization tools. From those, 7 tools were adapted to our problematic and 5 where fully operational: Phenolyzer^30^, Endeavour^17^, MaxLink^21^, ToppGene^18^, ToppNet^18^. To run each method, we used the list of deregulated urinary proteins as input and selected default parameters. The detailed parameters are available in Supplementary Data S2–S6.

#### Prioritization based on experimental results (Exp)

For prioritization based on experimental results, differentially abundant proteins from the four proteomics datasets were ranked based on their p-value (from smallest to largest).

#### Prioritization based on URA algorithm

Prioritization based on URA algorithm was performed using IPA software (content version release date 2017-12-07). This analysis examines how many known targets of each upstream regulator are present in the experimental dataset. Disease candidates (limited to proteins) were ranked based on the overlap p-value. The overlap p-value, calculated using Fisher’s Exact Test, measures whether there is a statistically significant overlap between the experimental dataset and the known targets that are under control of the upstream regulator.

### List of reference disease candidates

Disease candidates already referenced to be associated to ADPKD and UPJ were collected from Comparative Toxicogenomics Database (CTDbase) (http://ctdbase.org)^47^ using the R package CTDquerier^48^. CTDbase contains curated and inferred disease candidates. Curated candidates are extracted from the published literature by CTD curators or are derived from the OMIM database. The majority of disease candidates are inferred thought the association with a ‘drug and chemicals’ element (according to the MeSH definition^49^). A disease candidate is associated to the disease if a chemical compound or drug has an effect on the disease and on the expression of the gene. The more chemicals or drugs are associated with the disease and the disease candidate, the stronger is the association. For ADPKD, 504 disease candidates were found to be associated to the term “Polycystic Kidney, Autosomal Dominant”. For UPJ, 17,786 disease candidates were associated to the term “Ureteral Obstruction”. In order to obtain comparable results with ADPKD, we selected the first 500 reference disease candidates according to their inference score. Moreover, to assess overall specificity of the prioritization strategies, we also collected reference disease candidates from 80 other diseases (40 associated to the term “Urogenital disease” and 40 associated to other type of diseases) (Supplementary Table S5). For each of these diseases, we selected the first 500 reference disease candidates according to their inference score.

### Precision measurement

In order to evaluate the performance of PRYNT and the reference approaches, we compared the top 100 ranked candidates to the list of reference disease candidates obtained from CTDbase and calculated the precision of each method. The precision of the prioritization is the percentage of reference disease candidates in the ranking. The precision curve represents the precision depending on the size of the ranking taken into account. The area under the precision curve (AUC) was estimated using the trapezoidal rule.

### Specificity assessment

#### Cross-specificity

Cross-specificity was assessed by calculating the difference between precision AUC for reference specific disease candidates and precision AUC for reference non-specific disease candidates. A positive difference was expected to be associated with specific approach while a negative difference was expected to be in favor of a lack of specificity. For ADPKD datasets, specific precision AUC was calculated based on prioritization of reference ADPKD candidates, and non-specific AUC was calculated based on prioritization of reference UPJ candidates. Conversely, specific precision AUC for UPJ datasets was calculated based on prioritization of reference UPJ candidates, and non-specific AUC was calculated based on prioritization of reference ADPKD candidates.

#### Overall specificity

Overall specificity was assessed by ranking precision AUC for reference specific disease candidates and precision AUC of reference candidates from the 80 non-specific diseases. Specific prioritization method was expected to be associated with specific AUC being in the top ranked AUCs.

### Pathway enrichment analysis

KEGG pathway enrichment analysis^31^ was performed using the R package limma^50^. A pathway was considered associated to the set of candidates if its p-value was under 0.05.

### Systematic literature research

A systematic scientific literature search was performed to determine the link between the top 10 predicted proteins and the diseases of interests using the Google search engine by association of the different aliases of the top 10 proteins listed in Genecards with terms related to the disease. The terms used for ADPKD were: 'ADPKD', 'PKD1’, 'PKD2’ or 'polycystic kidney disease'. The terms chosen for the UPJ were: 'UPJ', ’ureteropelvic junction obstruction’, 'UUO model', and ‘ureteral obstruction'. The publications selected by this strategy were then analyzed manually to confirm the relevance of these studies linking a protein candidate to the disease of interest.

### Access to PRYNT

We developed an R interactive web application to improved accessibility of our method. Guide to getting started is available at: https://github.com/Boizard/PRYNT/tree/master/AppPRYNT.

## Supplementary Information

Supplementary Information.

## Data Availability

All data analyzed during this study are included in Bakun et al.^5^ (ADPKD1) (Supplementary Table S1); Rauniyar et al.^9^ (ADPKD2) (Supplementary Table S2); Lacroix et al.^7^ (UPJ1) (Supplementary Table S3); Chen et al.^6^ (UPJ2) (Supplementary Table S4). Data are also available on GitHub at: https://github.com/Boizard/PRYNT/tree/master/datas.

## Code availability

PRYNT’s source code is freely available on GitHub at: https://github.com/Boizard/PRYNT.
