# Supplementary material for: PRYNT: a tool for prioritization of disease candidates from proteomics data using a combination of shortest-path and random walk algorithms
Source: Sci Rep. 2021 Mar 11;11:5764. doi: 10.1038/s41598-021-85135-3 (PMC7952700; doi:10.1038/s41598-021-85135-3)
Supplement: Supplementary file 1 — Supplementary Information. [file 41598_2021_85135_MOESM1_ESM.pdf]

PRYNT: a tool for prioritization of disease candidates from proteomics data using a combination of shortest-path and random walk algorithms

Franck Boizard[1,2,+], Bénédicte Buffin-Meyer[1,2,+], Julien Aligon[3], Olivier Teste[4], Joost P. Schanstra[1,2,++], Julie Klein[1,2,++,*].

[1]Institut National de la Santé et de la Recherche Médicale (INSERM), U1048, Institut of Cardiovascular and Metabolic Disease, 31432 Toulouse, France

[2]Université Toulouse III Paul-Sabatier, 31330 Toulouse, France

[3]Université de Toulouse, UT1, IRIT, (CNRS/UMR 5505), Toulouse, France

[4]Université de Toulouse, UT2J, IRIT, (CNRS/UMR 5505), Toulouse, France

[+,++] Equal contribution

* julie.klein@inserm.fr

Supplementary material

Table of content

Supplementary Table S1. List of deregulated proteins from ADPKD1 dataset……………………………………………p3

Supplementary Table S2. List of deregulated proteins from ADPKD2 dataset……………………………………………p8

Supplementary Table S3. List of deregulated proteins from UPJ1 dataset…………………………………………………p11

Supplementary Table S4. List of deregulated proteins from UPJ2 dataset…………………………………………………p16

Supplementary Table S5. List of 80 diseases from CTDbase that were used to assess PRYNT overall specificity………p22

Supplementary Table S6. Literature search of the top 10 ranked by PRYNT for ADPKD…………………………p25

Supplementary Table S7. Literature search of the top 10 ranked by PRYNT for UPJ………………………………p26

Supplementary Figure S1. Performance of PRYNT compared to shortest-path and random walk algrotihms……p27

Supplementary Figure S2. Performance of PRYNT compared to common, state of the art prioritization strategies……p28

Supplementary Figure S3. Performance of PRYNT compared to reference approaches………p29

Supplementary Data S1. Pseudo-code of PRYNT algorithm………………………………………………………………p30

Supplementary Data S2. ToppGene's parameters…………………………………………………………………………p33

Supplementary Data S3. Phenolyzer's parameters………………………………………………………………………p34

Supplementary Data S4. Endeavour's parameters………………………………………………………………………p35

Supplementary Data S5. MaxLink's parameters…………………………………………………………………………p36

Supplementary Data 6. ToppNet's parameters……………………………………………………………………………p37

Supplementary references…………………………………………………………………………………………………p38

Supplementary Table 1. List of deregulated proteins from ADPKD1 dataset (Bakun et al. [1]).

	Gene symbol	ratio	q-value
**1**	A2M	3.16	3E-05
**2**	ADM	0.49	0.04219
**3**	AFM	1.7	3E-05
**4**	AGT	1.7	3E-05
**5**	ALB	1.69	3E-05
**6**	AMY1A	0.47	0.02277
**7**	AMY2A	0.7	0.00025
**8**	ANGPTL2	0.61	0.00801
**9**	ANPEP	0.71	0.0253
**10**	ANXA11	0.59	0.00632
**11**	APOA1	3.89	3E-05
**12**	APOA2	1.92	3E-05
**13**	APOA4	4.19	3E-05
**14**	APOB	3.34	3E-05
**15**	APOD	0.46	0.00013
**16**	APOE	0.69	0.04059
**17**	ART3	2.49	0.00569
**18**	AXL	0.62	0.0298
**19**	B2M	3.69	3E-05
**20**	B3GNT1	0.52	0.0032
**21**	B3GNT2	0.44	0.00271
**22**	BHMT	0.55	3E-05
**23**	BLMH	0.38	3E-05
**24**	C1RL	0.59	3E-05
**25**	C3	2.45	3E-05
**26**	C4B	1.4	3E-05
**27**	C5	1.9	0.01113
**28**	C7	2.02	0.03556
**29**	CA1	3.21	3E-05
**30**	CADM1	0.68	0.01829
**31**	CASP14	0.44	3E-05

**32**	CD14	0.64	0.00376
**33**	CD248	0.52	3E-05
**34**	CDH11	0.63	0.0019
**35**	CDH13	0.67	0.00212
**36**	CDH2	0.65	0.01885
**37**	CDH6	0.59	0.007
**38**	CDHR5	0.6	0.02658
**39**	CEL	0.54	3E-05
**40**	CETP	0.51	0.00632
**41**	CFB	1.66	0.00492
**42**	CFD	5.44	3E-05
**43**	CFH	1.65	0.01953
**44**	CHL1	0.51	3E-05
**45**	CILP	0.49	0.04059
**46**	CILP2	0.61	0.00569
**47**	CNTN1	0.69	0.00584
**48**	COL15A1	0.64	0.00029
**49**	COL6A1	0.53	3E-05
**50**	CPN2	0.64	0.01403
**51**	CRB2	0.51	0.01317
**52**	CRYAB	0.49	0.01487
**53**	CRYM	0.62	0.04647
**54**	CST6	1.85	0.00399
**55**	DPEP1	0.66	0.0143
**56**	DSC1	3.06	0.03104
**57**	EGF	0.59	3E-05
**58**	FAT4	0.51	0.00142
**59**	FBN1	0.72	0.00684
**60**	FBP1	0.75	0.02277
**61**	FCGR3B	0.6	0.04588
**62**	FGB	2.17	3E-05
**63**	FGG	2.89	3E-05

**64**	FLG	0.51	3E-05
**65**	FLRT2	0.59	0.00801
**66**	FN1	0.8	0.01028
**67**	FREM2	0.6	3E-05
**68**	FXYD2	0.4	0.00569
**69**	GAA	0.75	0.02277
**70**	GALNS	0.73	0.04062
**71**	GAS6	0.56	0.00553
**72**	GC	1.78	3E-05
**73**	GGCT	0.63	0.0075
**74**	GOLM1	0.52	0.00023
**75**	GP5	0.46	0.00467
**76**	GPC1	0.68	0.01447
**77**	GPC3	0.57	0.008
**78**	GPC4	0.72	0.0383
**79**	GPR116	0.57	0.02011
**80**	GSTM3	0.65	0.02672
**81**	GSTT1	0.4	0.04618
**82**	GUCA2B	3.75	0.01319
**83**	HP	2.21	3E-05
**84**	HPX	1.56	0.00083
**85**	HRG	1.81	8E-05
**86**	HSPB1	0.59	0.04848
**87**	HSPG2	0.75	0.01045
**88**	HYAL1	0.58	0.04254
**89**	ICAM2	0.63	0.03484
**90**	IGFBP6	5.08	3E-05
**91**	IGFBP7	0.45	3E-05
**92**	ISLR	0.63	0.0148
**93**	ITFG1	0.56	0.01951
**94**	ITIH1	1.39	0.00134
**95**	ITIH2	1.77	3E-05

**96**	KRT1	1.81	3E-05
**97**	KRT4	0.3	3E-05
**98**	KRT6A	0.57	0.00417
**99**	LCAT	0.56	3E-05
**100**	LGALS3BP	0.73	0.03793
**101**	LPHN1	0.6	0.01483
**102**	LRRC15	0.46	0.00411
**103**	MAN1A1	0.48	3E-05
**104**	MB	3.59	0.00175
**105**	MCAM	0.65	0.03723
**106**	MFI2	0.54	3E-05
**107**	MME	0.59	0.00049
**108**	MMRN2	0.58	0.00255
**109**	MUC20	0.44	3E-05
**110**	MXRA8	0.67	0.0055
**111**	MYOC	2.39	0.00134
**112**	OGN	2.5	0.01175
**113**	OLR1	0.56	0.00082
**114**	ORM1	1.94	3E-05
**115**	OSCAR	0.55	0.00221
**116**	PCDH1	0.65	0.02013
**117**	PCDHGC3	0.6	0.00038
**118**	PDCD6	0.56	0.02011
**119**	PFN1	2.42	0.00581
**120**	PHPT1	0.68	0.01598
**121**	PON1	3.48	0.01891
**122**	PROM2	0.65	0.02628
**123**	PSAP	2.74	3E-05
**124**	PSG11	5.92	0.00203
**125**	PSG9	5.04	0.00069
**126**	PVALB	0.52	0.03378
**127**	PVR	0.55	8E-05

**128**	PVRL1	0.6	0.02277
**129**	PVRL3	0.59	0.04733
**130**	QPCT	0.51	3E-05
**131**	QSOX1	0.72	0.01951
**132**	RBP4	2.65	3E-05
**133**	REG1A	1.7	0.00803
**134**	ROBO4	0.41	3E-05
**135**	RTN4RL2	0.61	0.00622
**136**	SDF4	0.58	0.00684
**137**	SDK1	0.61	0.01891
**138**	SERPINA1	1.64	3E-05
**139**	SERPINA4	0.57	0.00882
**140**	SERPINA5	0.38	3E-05
**141**	SERPINC1	2.01	3E-05
**142**	SERPIND1	2.32	3E-05
**143**	SERPINF1	2.51	3E-05
**144**	SERPINI1	0.59	0.00082
**145**	SH3BGRL	2.03	0.00794
**146**	SPRR3	0.49	6E-05
**147**	SUSD2	0.66	0.02173
**148**	TF	1.84	3E-05
**149**	TGM4	0.67	0.01365
**150**	TNXB	0.75	0.00569
**151**	TPP1	0.69	0.04437
**152**	TTR	1.88	8E-05
**153**	VASN	0.46	3E-05
**154**	XPNPEP2	0.68	0.04733

Supplementary Table 2. List of deregulated proteins from ADPKD2 dataset (Rauniyar et al. [2]).

	Gene symbol	log2FC	p-value
**1**	A1BG	2.46	0.00101
**2**	ABHD14B	1.61	0.00799
**3**	AFM	3.82	0.0036
**4**	AGT	3.55	0.00122
**5**	AHSG	1.56	0.01839
**6**	ALB	3.49	0.00175
**7**	AMBP	2.18	0.00063
**8**	APOA1	3.79	0.0162
**9**	APOA2	3.21	0.01466
**10**	APOA4	4.71	0.00535
**11**	ARSF	-1.19	0.01498
**12**	AZGP1	1.99	0.00029
**13**	C1orf123	2.9	0.02426
**14**	C3	3.63	0.02694
**15**	C9	3.38	0.00438
**16**	CA1	3.95	0.03413
**17**	CHGA	4.04	0.01849
**18**	CP	2.65	0.00178
**19**	CTSC	1.98	1E-05
**20**	CTSD	2.14	0
**21**	CTSZ	2.33	0.00034
**22**	DPEP1	-1.4	0.00012
**23**	DPP7	1.34	0.00069
**24**	DSC2	-3.02	0.01076
**25**	EGF	-2.05	0
**26**	ENDOD1	1.24	0.00034
**27**	FCN2	1.24	0.01735
**28**	FTH1	1.31	0.00865
**29**	FTL	2.48	0.00164
**30**	GAS6	-1.79	1E-05

**31**	GC	3.49	0.00459
**32**	GDF15	1.43	0.00265
**33**	GM2A	2.07	0.0002
**34**	GP1BA	1.6	0.0021
**35**	GPX3	1.03	0.00724
**36**	GSN	1.6	0.00119
**37**	GUCA2A	2.78	0.03151
**38**	HP	2.85	0.01144
**39**	HPX	3.41	0.00203
**40**	IGKC	1.63	0.00054
**41**	IGLV3-19	2.62	0.01567
**42**	KLK1	-1.94	0.00024
**43**	LCN1	1.69	0.00262
**44**	LRG1	2.41	4E-05
**45**	LRRC15	-1.2	0.0023
**46**	LTBP1	1.17	0.01944
**47**	LYVE1	1.46	8E-05
**48**	MSLN	2.47	0.00167
**49**	OMD	-1.24	0.00276
**50**	ORM2	2.83	0.00036
**51**	PAPPA2	2.96	0
**52**	PI16	1.67	0.00189
**53**	PIP	-1.2	0.01125
**54**	PLAU	-1.38	0.00421
**55**	PLG	2.59	0.00362
**56**	RBP4	4.4	0.01903
**57**	RNASET2	1.75	0.00073
**58**	SERPINA1	3.58	0.00275
**59**	SERPINA3	2.44	0.00195
**60**	SERPINA6	1.96	0.00223
**61**	SERPINC1	3.68	0.00339
**62**	SERPIND1	2.75	0.02005

**63**	SERPINF1	4.22	0.01026
**64**	SOD1	1.76	0.00701
**65**	SPINK5	3.12	0.01279
**66**	TF	3.7	0.00181
**67**	TFF1	2.72	1E-05
**68**	VCAM1	1.75	0.00011
**69**	XPNPEP2	-1.35	0.00013

Supplementary Table 3. List of deregulated proteins from UPJ1 dataset (Lacroix et al. [3]).

	Gene symbol	ratio	p-value
**1**	A1BG	3.78104	0.04295
**2**	A2M	24.53253	0.00449
**3**	A2ML1	-845.56074	0.00203
**4**	ACAN	-2.38691	0.00798
**5**	ACTB	2.33965	0.02911
**6**	ACTG1	2.38691	0.029
**7**	AFM	2.82922	0.00512
**8**	AGT	15.33289	0.00408
**9**	AHSG	3.38719	0.01068
**10**	ALB	14.58509	7E-05
**11**	ALB	14.87973	0.00012
**12**	ALB	15.02928	0.00012
**13**	AMY1B	-7.31553	0.016
**14**	AMY2B	-7.46332	0.01819
**15**	APOA1	42.09799	0.00559
**16**	APOA1	36.59823	0.00673
**17**	APOA2	64.07152	0.00375
**18**	APOA4	15.79984	0.01292
**19**	APOA4	8.16617	0.0417
**20**	APOE	9.97418	0.00067
**21**	APP	-2.36316	0.03879
**22**	ARG1	-11.70481	0.00478
**23**	ASAH1	1.97388	0.00117
**24**	AXL	-1.95424	0.00483
**25**	B2M	2.07508	0.03824
**26**	BLMH	-10.27794	0.00567
**27**	BMPR2	-4.0552	0.04912
**28**	BSG	-21.11534	0.01453
**29**	C3	24.53253	0.00107
**30**	C4A	26.04954	0.02095

**31**	C4B	18.54129	0.01865
**32**	C5	55.70111	0.04118
**33**	CADM1	-1.84043	0.00959
**34**	CADM4	-1.23368	0.0301
**35**	CALML5	-48.42422	0.00373
**36**	CAPS	60.34029	0.00245
**37**	CASP14	-3.52542	0.01227
**38**	CD300A	-1.85893	0.03843
**39**	CD44	-2.53451	0.0021
**40**	CD9	-2.53451	0.03073
**41**	CDH1	1.97388	0.02542
**42**	CDH6	1.97388	0.01392
**43**	CDH8	-11.58835	0.00011
**44**	CFB	9.58309	0.0026
**45**	CFH	11.02318	0.0022
**46**	CFHR2	10.48557	0.02722
**47**	CFI	2.69123	0.04509
**48**	COL3A1	10.8049	0.04699
**49**	CP	6.4883	0.0179
**50**	CSPG4	-2.03399	0.04014
**51**	CST3	20.49129	0.00171
**52**	CTSZ	2.85765	0.00682
**53**	CUBN	-1.41907	0.03059
**54**	DAG1	-19.29797	0.03142
**55**	DDR1	21.97708	0.00076
**56**	DLST	3.49034	0.00858
**57**	DMBT1	-28.50273	0.00171
**58**	DSG1	15.48699	0.0343
**59**	ECM1	9.39333	0.00054
**60**	ECM1	9.77668	0.00086
**61**	EGF	-3.9749	0.01171
**62**	ENSG00000244255	17.11577	0.00108

**63**	EPHA7	-3.9749	0.04195
**64**	FAM174A	-3.56085	0.03756
**65**	FBN1	-3.89619	0.03444
**66**	FCER2	-43.81604	0.00887
**67**	FCGR3A	-5.41948	0.00315
**68**	FCGR3B	-4.13712	0.01645
**69**	FGB	30.26524	0.04757
**70**	FGG	102.51406	0.01705
**71**	FLG	-5.00281	0.00767
**72**	FLNB	2.2705	0.00833
**73**	GC	13.46374	0.00394
**74**	GGCT	-15.48699	0.00063
**75**	GGT1	-22.42104	0.0002
**76**	GNAL	-1.64872	0.02509
**77**	GSN	3.89619	0.0091
**78**	HABP2	59.73989	0.01216
**79**	HBA2	138.37951	0.02475
**80**	HBB	12.18249	0.01083
**81**	HBD	6.42374	0.00614
**82**	HPX	6.95875	0.00672
**83**	HRNR	2.03399	0.03908
**84**	HSPA8	10.48557	0.00021
**85**	HSPA8	9.48774	0.00029
**86**	HSPB1	52.45733	0.01553
**87**	IBSP	2.01375	0.0174
**88**	ICOSLG	-2.24791	0.01123
**89**	IFNAR2	-35.87354	0.00539
**90**	IGFBP7	-1.53726	0.03826
**91**	IL2RA	-3.35348	0.03512
**92**	ITIH1	13.19714	0.0266
**93**	ITIH2	159.17433	0.00068
**94**	IVL	29.9641	0.00603

**95**	KIRREL	5.15517	0.00039
**96**	KLK1	-23.33606	0.02004
**97**	KRT7	99.48432	0.02205
**98**	KRT8	132.95357	0.00955
**99**	LAIR1	-2.22554	0.02701
**100**	LAIR1	-2.05443	0.03061
**101**	LAMC1	-7.53832	0.00015
**102**	LPA	-25.02812	4E-05
**103**	LRG1	3.85743	0.01494
**104**	LRP2	-2.80107	0.00041
**105**	LRP2	-2.43513	0.00296
**106**	LRRC15	-28.50273	2E-05
**107**	LUM	4.1787	0.01598
**108**	LUM	4.13712	0.01882
**109**	MME	-17.28778	0.00092
**110**	MSLN	-1.63232	0.04532
**111**	MSN	7.17068	0.01188
**112**	MYL6	8.08492	0.01521
**113**	NCAN	-3.9749	0.00148
**114**	OMD	2.48432	0.0275
**115**	PDCD1LG2	-2.85765	0.00427
**116**	PODXL	-4.4371	0.00787
**117**	PROM2	-11.58835	0.02648
**118**	PRR11	19.49192	0.00053
**119**	PTGDS	6.17186	0.01767
**120**	PTK7	3.32012	0.00128
**121**	PVR	-1.75067	0.00951
**122**	RBP4	3.6693	0.00997
**123**	RGL4	12.93582	0.00623
**124**	S100A7	-20.90524	0.04458
**125**	S100P	8.08492	0.02368
**126**	SERPINA1	16.60992	0.00337

**127**	SERPINA3	7.7679	0.02501
**128**	SERPINA5	-9.97418	0.01169
**129**	SERPINB3	-43.38006	0.00128
**130**	SERPINC1	11.94126	0.00919
**131**	SGSH	-12.18249	0.0297
**132**	SNCG	14.87973	0.04558
**133**	SORL1	-10.07442	0.00039
**134**	TF	68.03348	0.00253
**135**	TGM3	-64.07152	0.0002
**136**	THBD	-1.71601	0.00807
**137**	THBD	-1.66529	0.00782
**138**	TPM3	4.34924	0.02219
**139**	TPM3	5.25931	0.02845
**140**	TTR	12.30493	0.01343
**141**	TUBA1C	16.94546	0.00554
**142**	VCAN	4.85496	0.03098
**143**	VCAN	4.71147	0.03184
**144**	VPS4B	-7.61409	0.0001
**145**	VTN	1.58407	0.01743

Supplementary Table 4. List of deregulated proteins from UPJ2 dataset (Chen et al. [4]).

	Gene symbol	ratio	p-value
**1**	A2M	2.5549	0.0004-
**2**	A2ML1	0.7301	0.0015
**3**	ABP1	0.768	0.0002
**4**	ACPP	0.624	0
**5**	ACTA2	2.0307	0
**6**	ACTB	1.6439	0
**7**	ADH5	1.7459	0.0142
**8**	AFM	1.8763	0.0067
**9**	AGT	1.9072	0
**10**	AHSG	2.3831	0.0001
**11**	ALDOB	0.7178	0.0025
**12**	AMBP	1.5326	0.0073
**13**	AMY2A	0.6454	0.0002
**14**	APCS	1.6602	0.0071
**15**	APOB	2.0665	0.0042
**16**	APOH	1.6455	0.024
**17**	ART3	0.75	0.0006
**18**	BHMT	0.638	0.0133
**19**	C3	2.4947	0
**20**	C4B	1.8248	0.0005
**21**	C5	2.2294	0.0065
**22**	CA1	2.0388	0.0335
**23**	CADM1	0.7437	0
**24**	CADM4	0.7536	0
**25**	CAP1	1.6467	0.0014
**26**	CAPG	1.6412	0.0005
**27**	CD300LG	2.7834	0.0077
**28**	CD33	0.7158	0.0062
**29**	CD7	0.7266	0
**30**	CD93	1.5976	0.0033

**31**	CD99L2	0.7078	0.0069
**32**	CFB	1.9627	0
**33**	CFH	1.5206	0.0018
**34**	CFHR1	1.5937	0.0226
**35**	CFI	1.9078	0.0007
**36**	CHGB	0.5093	0
**37**	CHMP5	0.5473	0.0001
**38**	CLIC1	1.6591	0.0006
**39**	CLN5	1.3351	0.0038
**40**	CLU	2.0272	0.0028
**41**	CNDP2	1.4015	0.0252
**42**	COCH	0.7523	0.0015
**43**	COL2A1	0.7231	0.0044
**44**	COL6A1	1.4365	0.006
**45**	COMP	0.7638	0.0007
**46**	CP	1.7465	0.0015
**47**	CTSC	1.3671	0.0093
**48**	CX3CL1	0.7349	0.0318
**49**	DCD	0.768	0.0048
**50**	DLK1	1.8776	0.0009
**51**	DNER	0.6936	0.0001
**52**	DPEP1	0.767	0.0015
**53**	DSPP	0.7012	0.0027
**54**	EEF1A1	1.5971	0
**55**	EFEMP1	1.317	0.0155
**56**	EHD4	1.4136	0.0002
**57**	FAIM3	0.6041	0.0001
**58**	FAM198B	0.7647	0.0055
**59**	FASN	1.4224	0.0223
**60**	FCAMR	0.5681	0
**61**	FGA	2.3488	0.0001
**62**	FGB	2.473	0.0002

**63**	FGG	2.6412	0.0001
**64**	FLG	0.4976	0
**65**	FOLR1	1.4144	0.0137
**66**	FSTL1	1.5256	0.0016
**67**	GALNS	0.7657	0.0135
**68**	GGCT	0.5455	0
**69**	GSN	1.5695	0.001
**70**	GSTM3	1.3972	0
**71**	HBA2	2.8766	0.0004
**72**	HBB	1.6876	0.004
**73**	HBG1	4.2334	0.0001
**74**	HIST1H1E	1.7724	0.0072
**75**	HIST1H2BK	2.1685	0.0001
**76**	HIST1H3F	1.9323	0.0146
**77**	HIST1H4A	1.4952	0.011
**78**	HP	2.5651	0.0164
**79**	HPX	2.036	0.0001
**80**	HSP90AA1	1.4013	0.0014
**81**	HSPB1	1.408	0.0022
**82**	ITIH1	1.9939	0.0084
**83**	ITIH2	1.6485	0.0031
**84**	ITLN1	0.6814	0.0009
**85**	KLK1	0.4488	0.0001
**86**	KLK8	0.7377	0.015
**87**	LAMA4	0.7358	0.0009
**88**	LGALS9B	0.6435	0.0011
**89**	LINGO1	0.731	0.0004
**90**	LPA	0.5373	0.0057
**91**	LRG1	1.6554	0.0276
**92**	LRRN4	2.3819	0.0147
**93**	LTBP2	0.7521	0.0206
**94**	LYPD3	0.6201	0.0001

**95**	LYVE1	1.6135	0.0005
**96**	MMP7	4.2294	0
**97**	MSLN	1.3821	0.0083
**98**	MYH9	1.5973	0.0002
**99**	MYO1C	1.3309	0.0172
**100**	NBL1	1.6532	0.0081
**101**	NPEPPS	1.7566	0.0135
**102**	NRP1	1.9719	0.0225
**103**	NUTF2	0.4002	0.0195
**104**	OPCML	0.7123	0
**105**	ORM1	2.4135	0.0004
**106**	ORM2	2.1989	0.0005
**107**	PAPPA2	1.5268	0.0004
**108**	PCDHGC3	0.7434	0.001
**109**	PGLYRP1	0.7379	0.0003
**110**	PI16	1.6405	0.0067
**111**	PKM	1.3576	0.0082
**112**	PLS3	0.7539	0.0056
**113**	PPIA	1.6195	0.0001
**114**	PPIC	1.5275	0.0102
**115**	PRND	2.0571	0.0023
**116**	PROM1	1.3774	0.0009
**117**	PROM2	0.7689	0.0013
**118**	PROS1	1.7832	0.001
**119**	PSCA	0.6753	0.0112
**120**	PTGDS	1.5082	0.008
**121**	PTPRN	0.6437	0
**122**	PTPRZ1	0.6541	0.0032
**123**	REG1A	0.7653	0.0015
**124**	RNASET2	1.3859	0.0004
**125**	ROR2	0.5393	0.0172
**126**	SCUBE2	1.4695	0.0025

**127**	SECTM1	1.5237	0.0018
**128**	SERPINA1	2.0147	0.0001
**129**	SERPINA3	1.7338	0.0008
**130**	SERPINA4	1.6769	0.0006
**131**	SERPINA7	1.3429	0.0024
**132**	SERPINB12	0.5751	0.0153
**133**	SERPINB3	0.5726	0.0003
**134**	SERPINB4	0.6144	0.0003
**135**	SERPINC1	2.1689	0
**136**	SERPIND1	1.9181	0.0001
**137**	SERPINF2	1.5341	0
**138**	SERPING1	1.7187	0.0029
**139**	SEZ6L	0.7348	0.0038
**140**	SHISA6	0.6578	0.0016
**141**	SHISA7	0.6711	0.0013
**142**	SPARC	1.4356	0.0115
**143**	SPP1	2.1167	0.0118
**144**	ST13	1.6829	0.0093
**145**	TF	3.1592	0
**146**	TGM3	0.5203	0.0037
**147**	TGOLN2	0.7413	0
**148**	TIMP1	2.005	0.0068
**149**	TTR	1.5459	0.0285
**150**	TUBA1A	1.7014	0
**151**	TUBA4A	1.7037	0.0001
**152**	TUBB	1.9216	0.0012
**153**	TUBB4B	1.4499	0.0006
**154**	TWSG1	1.5528	0.0024
**155**	UMOD	1.5087	0.0071
**156**	VMO1	0.7623	0.0032
**157**	VPS4B	0.7474	0.0048
**158**	VTCN1	1.9246	0.0008

**159**	VTN	1.3744	0.0035
**160**	WFDC2	2.5363	0.0094
**161**	XYLT1	0.5797	0.0002
**162**	YWHAZ	2.1473	0.0024

Supplementary Table 5. List of 80 diseases from CTDbase that were used to assess PRYNT overall specificity.

**DiseaseName**	**DiseaseID**	SlimMappings
Candidiasis, Invasive	MESH:D058365	Bacterial infection or mycosis
Tuberculosis, Gastrointestinal	MESH:D014385	Bacterial infection or mycosis|Digestive system disease
Tuberculosis, Pleural	MESH:D014396	Bacterial infection or mycosis|Respiratory tract disease
Leukemia, Myeloid, Chronic-Phase	MESH:D015466	Blood disease|Cancer
Colorectal Neoplasms, Hereditary Nonpolyposis	MESH:D003123	Cancer|Digestive system disease|Genetic disease (inborn)|Metabolic disease
Lymphocele	MESH:D008210	Cancer|Lymphatic disease
Aortitis	MESH:D001025	Cardiovascular disease
Embolism, Cholesterol	MESH:D017700	Cardiovascular disease
Microvascular Angina	MESH:D017566	Cardiovascular disease
Anus, Imperforate	MESH:D001006	Congenital abnormality|Digestive system disease
Acrocephalosyndactylia	MESH:D000168	Congenital abnormality|Musculoskeletal disease
Alzheimer disease type 2	MESH:C536595	Mental disorder|Nervous system disease
Contracture	MESH:D003286	Musculoskeletal disease
Osteosclerosis	MESH:D010026	Musculoskeletal disease
Fatigue Syndrome, Chronic	MESH:D015673	Musculoskeletal disease|Nervous system disease|Viral disease
Brachial Plexus Neuritis	MESH:D020968	Nervous system disease
Child Nutrition Disorders	MESH:D015362	Nutrition disorder
Eclampsia	MESH:D004461	Pregnancy complication
Facial Dermatoses	MESH:D005148	Skin disease
Arterivirus Infections	MESH:D018174	Viral disease
Intraabdominal Infections	MESH:D059413	Bacterial infection or mycosis
Tuberculosis	MESH:D014376	Bacterial infection or mycosis
Fibrosarcoma	MESH:D005354	Cancer
Leukemia, T-Cell	MESH:D015458	Cancer|Immune system disease|Lymphatic disease
Precursor T-Cell Lymphoblastic Leukemia-Lymphoma	MESH:D054218	Cancer|Immune system disease|Lymphatic disease
Skull Neoplasms	MESH:D012888	Cancer|Musculoskeletal disease
Endocardial Cushion Defects	MESH:D004694	Cardiovascular disease|Congenital abnormality
Hypoplastic Left Heart Syndrome	MESH:D018636	Cardiovascular disease|Congenital abnormality
Cerebral Arterial Diseases	MESH:D002539	Cardiovascular disease|Nervous system disease
Spina Bifida Occulta	MESH:D016136	Congenital abnormality|Nervous system disease
Gastritis	MESH:D005756	Digestive system disease
Intestinal Obstruction	MESH:D007415	Digestive system disease
Reye Syndrome	MESH:D012202	Digestive system disease|Metabolic disease|Nervous system disease
Hashimoto Disease	MESH:D050031	Endocrine system disease
Diabetic Neuropathies	MESH:D003929	Endocrine system disease|Nervous system disease
Graft vs Host Disease	MESH:D006086	Immune system disease
Encephalomyelitis, Autoimmune, Experimental	MESH:D004681	Immune system disease|Nervous system disease
Stress Disorders, Post-Traumatic	MESH:D013313	Mental disorder
Migraine without Aura	MESH:D020326	Nervous system disease
Neuromuscular Junction Diseases	MESH:D020511	Nervous system disease
Gonorrhea	MESH:D006069	Bacterial infection or mycosis|Urogenital disease (female)|Urogenital disease (male)

Pyuria	MESH:D011776	Bacterial infection or mycosis|Urogenital disease (female)|Urogenital disease (male)
Tuberculosis, Renal	MESH:D014398	Bacterial infection or mycosis|Urogenital disease (female)|Urogenital disease (male)
Tuberculosis, Urogenital	MESH:D014401	Bacterial infection or mycosis|Urogenital disease (female)|Urogenital disease (male)
Atypical Hemolytic Uremic Syndrome	MESH:D065766	Blood disease|Urogenital disease (female)|Urogenital disease (male)
Testicular Germ Cell Tumor	MESH:C563236	Cancer|Endocrine system disease|Urogenital disease (male)
Turner Syndrome	MESH:D014424	Cardiovascular disease|Congenital abnormality|Endocrine system disease|Genetic disease (inborn)|Urogenital disease (female)|Urogenital disease (male)
Disorder of Sex Development, 46,XY	MESH:D058490	Congenital abnormality|Endocrine system disease|Urogenital disease (female)|Urogenital disease (male)
Ovotesticular Disorders of Sex Development	MESH:D050090	Congenital abnormality|Endocrine system disease|Urogenital disease (female)|Urogenital disease (male)
Penile Induration	MESH:D010411	Connective tissue disease|Urogenital disease (male)
Vaginal Fistula	MESH:D014624	Pathology (anatomical condition)|Urogenital disease (female)
Vesicovaginal Fistula	MESH:D014719	Pathology (anatomical condition)|Urogenital disease (female)
Urinary Incontinence, Stress	MESH:D014550	Signs and symptoms|Urogenital disease (female)|Urogenital disease (male)
Endometritis	MESH:D004716	Urogenital disease (female)
Leukorrhea	MESH:D007973	Urogenital disease (female)
Uterine Cervicitis	MESH:D002575	Urogenital disease (female)
Balkan Nephropathy	MESH:D001449	Urogenital disease (female)|Urogenital disease (male)
Vesico-Ureteral Reflux	MESH:D014718	Urogenital disease (female)|Urogenital disease (male)
Hemospermia	MESH:D051516	Urogenital disease (male)
SPERMATOGENIC FAILURE 6	OMIM:102530	Urogenital disease (male)
Hemolytic-Uremic Syndrome	MESH:D006463	Blood disease|Urogenital disease (female)|Urogenital disease (male)
Carcinoma, Endometrioid	MESH:D018269	Cancer|Endocrine system disease|Urogenital disease (female)
Carcinoma, Ovarian Epithelial	MESH:D000077216	Cancer|Endocrine system disease|Urogenital disease (female)
Wilms Tumor	MESH:D009396	Cancer|Genetic disease (inborn)|Urogenital disease (female)|Urogenital disease (male)
46, XX Disorders of Sex Development	MESH:D058489	Congenital abnormality|Endocrine system disease|Urogenital disease (female)|Urogenital disease (male)
Hyperandrogenism	MESH:D017588	Congenital abnormality|Endocrine system disease|Urogenital disease (female)|Urogenital disease (male)
Cryptorchidism	MESH:D003456	Congenital abnormality|Endocrine system disease|Urogenital disease (male)
Diabetic Nephropathies	MESH:D003928	Endocrine system disease|Urogenital disease (female)|Urogenital disease (male)
Renal hypouricemia	MESH:C537757	Genetic disease (inborn)|Metabolic disease|Pathology (anatomical condition)|Urogenital disease (female)|Urogenital disease (male)
Fanconi Syndrome	MESH:D005198	Genetic disease (inborn)|Metabolic disease|Urogenital disease (female)|Urogenital disease (male)
Nocturnal Enuresis	MESH:D053206	Mental disorder|Urogenital disease (female)|Urogenital disease (male)

Nephrocalcinosis	MESH:D009397	Metabolic disease|Urogenital disease (female)|Urogenital disease (male)
Urinary Bladder, Neurogenic	MESH:D001750	Nervous system disease|Signs and symptoms|Urogenital disease (female)|Urogenital disease (male)
Azotemia	MESH:D053099	Pathology (process)|Urogenital disease (female)|Urogenital disease (male)
Albuminuria	MESH:D000419	Signs and symptoms|Urogenital disease (female)|Urogenital disease (male)
Oliguria	MESH:D009846	Signs and symptoms|Urogenital disease (female)|Urogenital disease (male)
Anuria	MESH:D001002	Urogenital disease (female)|Urogenital disease (male)
Kidney Diseases, Cystic	MESH:D052177	Urogenital disease (female)|Urogenital disease (male)
Nephrolithiasis	MESH:D053040	Urogenital disease (female)|Urogenital disease (male)
Urinary Retention	MESH:D016055	Urogenital disease (female)|Urogenital disease (male)

Supplementary Table 6. Literature search of the top 10 ranked by PRYNT for ADPKD.

Protein symbol#	Rank ADPKD1	Rank ADPKD2	Reference
**AKT1**	6	6	[5,6]
**CDH1**	383	5	[7,8 9]
**CTNNB1**	3	11	[10,11]
**F2**	2	2	-
**HSP90AA1**	11	7	[12]
**HSPA8**	15	8	-
**INS**	4	4	[13,14]
**JUN**	1	22	[15-17]
**MST1**	21	9	[18,19]
**MYB**	8	3	[20]
**NOTCH1**	43	10	[21,22]
**SP1**	7	18	[23,24]
**SRC**	10	27	[25,26]
**TP53**	9	1	[27,28 29]
**YY1**	5	*	[24]

#Uniprot protein symbol
*Not ranked

Supplementary Table 7. Literature search of the top 10 ranked by PRYNT for UPJ.

Protein symbol#	Rank UPJ1	Rank UPJ2	Reference
**ABL1**	10	*	-
**AKT1**	111	2	[30,31]
**CCNB1**	*	1	-
**CDH1**	336	3	[32]
**CDK1**	8	*	[33]
**F2**	3	6	-
**HDAC1**	*	7	[34]
**HSP90AA1**	6	81	[35]
**ITGB1**	*	4	[36,37]
**JUN**	5	20	[38,39]
**MAPK1**	2	11	[40,41]
**SRC**	9	8	[33]
**TNF**	4	23	[42-45]
**TP53**	1	12	[46,47]

#Uniprot protein symbol
*Not ranked

Supplementary Figure 1. Performance of PRYNT compared to shortest-path and random walk algorithms. The precision was calculated based on the percentage of reference ADPKD or UPJ disease candidates that were prioritized in the top 100 candidates ranked by the different strategies in the four datasets. SP: shortest-path; RW: random walk.

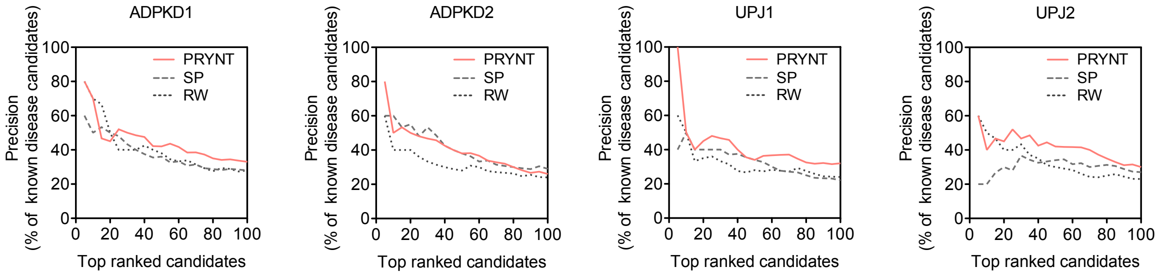

Supplementary Figure 2. Performance of PRYNT compared to common, state of the art prioritization strategies. The precision was calculated based on the percentage of reference ADPKD or UPJ disease candidates that were prioritized in the top 100 candidates ranked by the different strategies in the four datasets. D: direct; ICN+RW: interconnectedness combined with random walk.

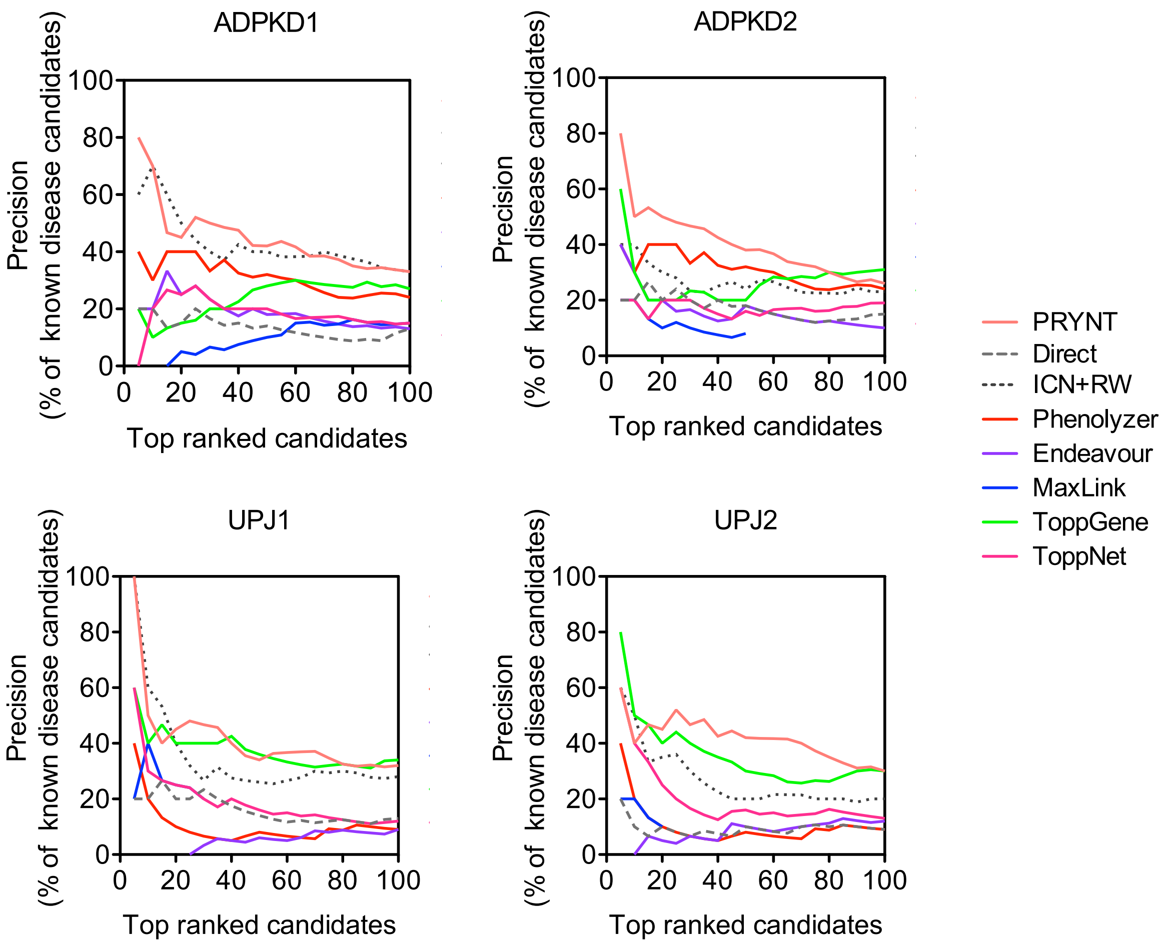

Supplementary Figure 3. Performance of PRYNT compared to reference approaches. The precision was calculated based on the percentage of reference ADPKD or UPJ disease candidates that were prioritized in the top 100 candidates ranked by the different strategies in the four datasets. Exp: experimental; URA: upstream regulator analysis.

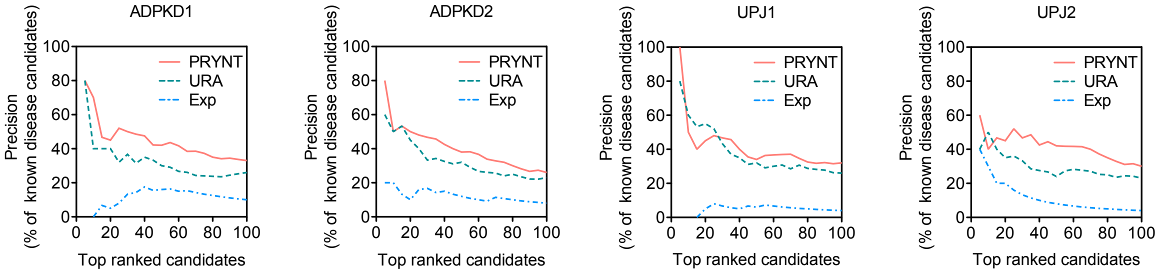

Supplementary Data 1. Pseudo-code of PRYNT algorithm.

ALGORITHM PRYNT is

INPUT
G = (V; E) *// we used the string network ("9606.protein.actions.v10.5.txt" download from string-db.org)*

- V = { V_1, …, V_n },
- E = { e_k | e_k = (V_i, V_j, P_k) where (V_i, V_j) an ordered pair, $V_i \in V$, $V_j \in V$, and P_k denotes three properties of the edge such as P_k.confidence, P_k.action and P_k.direction }.

P = { P_1,…, P_m } $\subseteq$ V *// set of deregulated protein (DP) from proteomic study*

OUTPUT
R = { (V_i, $score_i$) | $V_i \in V$, $score_i \in R^+$}

BEGIN
// Contextualisation of the graph
// selection of significant edges
select ← ∅
FOR $\forall e_k \in E$ **DO**
    IF P_k.confidence >= 0.9 **AND** P_k.action = "acting" **AND** P_k.direction = "directional" **THEN**
        select ← select ∪ { e_k }
    ELSE *// selection of edges connected to protein DP regardless of their scores*
    IF ($e_k.V_i \in P$ **OR** $e_k.V_j \in P$) **AND** P_k.action = "acting" **AND** P_k.direction = "directional" THEN
        select ← select ∪ { e_k }
    END IF
END FOR

SG ← (SV, select) | $\forall e_k \in$ select, SV←SV∪{$e_k.V_i$, $e_k.V_j$}

// set of cliques from SG (a clique is a group of vertices all connect to each other)
C = {C_1, …, C_g} | $\forall h \in$[1..g], C_h={ V_1,…, V_{ch} } | $V_i \in SV$, $V_j \neq V_i \in SV$, $\forall(V_i, V_j) \in C_h$, $\exists e_k=(V_i, V_j, P_k) \in$ select

G' ← SG | G' = (V', E') *// Graph containing vertices or clique as node*
P' ← P // *set of deregulated node containing vertices or clique*

FOR $\forall V_i \in SV$ **DO**
    bigger_cliques[i] ← ∅
    size_clique ← 2
    FOR $\forall C_h \in C$ **DO**
        IF $V_i \in C_h$ **AND** $|C_h|$>size_clique **THEN**
            size_clique ← $|C_h|$
            bigger_cliques[i] ← {C_h}
        ELSE
        IF $V_i \in C_h$ **AND** $|C_h|$=size_clique **THEN**
            bigger_cliques[i] ← bigger_cliques[i] ∪ {C_h}
        END IF
    END FOR
END FOR

sorted_bigger_cliques ← sort(bigger_cliques) *// Sort (or order) a vector from largest to smallest (R function)*
FOR $\forall$i∈1..|sorted_bigger_cliques| **DO**
    $\beta \leftarrow U_{h=1}^{|\text{sorted\_bigger\_cliques[i]}|} C_h$ *// set of node in the bigger.clique*
    V' ← V' ∪ {V_{new}} // *add new node representing the clique*
    E' ← E' ∪ { (V_{new}, V_j, P_k) | $\exists e_k.V_i \in \beta$, $e_k.V_j \notin \beta$ } // *add right external edges from the clique*
    E' ← E' ∪ { (V_i, V_{new}, P_k) | $\exists e_k.V_i \notin \beta$, $e_k.V_j \in \beta$ } // *add left external edges from the clique*

V' ← V' \ β // *delete nodes*
E' ← E' \ { e_k | $e_k.V_i$∈β ∨ $e_k.V_j$∈β } // *delete edges*
hash[V_{new}] ← β // *hash function structure where Vnew is used as an argument to access to β*

deregulated ← false
j ← 1
WHILE NOT deregulated **AND** j≤|β| **DO**
IF Vj∈P **THEN**
deregulated ← true
P' ← P' ∪ {V_{new}} \ β
END IF
END WHILE
END FOR

// Prioritization of nodes in the graph containing vertices or clique
// Shortest-path algorithm
FOR ∀V'_i∈V' **DO**

$$score[i] \leftarrow \frac{1}{\sum_{l=1}^{P'} \text{shortest\_path}\left(V'_i, P'_l\right)}$$

END FOR

FOR ∀V'_i∈V' **DO**
SP'[i] ← (V_i, *score*[i], *rank*(*score)*[i])) // *rank function of R returns the sample ranks of the values in a vector. In case of equal score ranks is replaces by their mean*
END FOR

// Random walk algorithm
A' ← compute.adjacency.matrix (G') *// function of RandomWalkRestartMH package*
score ← Random.Walk.Restart.Multiplex(A', P') *// function of RandomWalkRestartMH package*

FOR ∀V_i∈V **DO**
RW[i] ← (V_i, score[i], rank(score)[i])) // *rank function of R returns the sample ranks of the values in a vector. In case of equal score ranks is replaces by their mean*
END FOR

\\ *combined score*
FOR ∀V_i∈V' **DO**
CS'[i] ← SP'[i].rank * RW'[i].rank
END FOR

\\ *Calculation of priotization score in the selected graph, SG, in order to select the best vertice in each clique*
\\ *Shortest-path algorithm*
FOR ∀V_i∈V **DO**

$$score[i] \leftarrow \frac{1}{\sum_{l=1}^{P} \text{shortest\_path}\left(V_i, P_l\right)}$$

END FOR

FOR ∀V_i∈V **DO**
SP[i] ← (V_i, *score*[i], *rank*(*score)*[i]))
END FOR

\\ *Random walk algorithm*
A ← compute.adjacency.matrix (G) *// function of RandomWalkRestartMH package*
score ← Random.Walk.Restart.Multiplex(A, P) *// function of RandomWalkRestartMH package*

```
FOR ∀Vi∈V DO
        RW[i] ← (Vi, score[i], rank(score)[i])) // rank function of R returns the sample ranks of the values in a vector.
In case of equal score ranks is replaces by their mean
END FOR

\\ combined score
FOR ∀Vj∈SV DO
        CS[j] ← SP[j].rank * RW[j].rank
END FOR

\\ For any vertice being a clique in the G' graph, we select the vertice included in the clique with the best score in the G
graph as a representative.
FOR ∀Vi∈V' DO
        IF Vi∈V THEN
                R[i] ← (Vi, score'[i])
        ELSE // Vi represents a set of bigger cliques
                min_score ← n^2 // initialisation of min score. n is the number of node in the G  graphe, so the rank
couldn't be larger than n and the score no more than n*n
                FOR ∀Vj∈SV DO
                        IF Vj∈hash[Vnew] AND score[j] < min_score THEN
                                R[i] ← (Vj, score'[i])
                                min_score ← score[j]
                        END IF
                END FOR
        END IF
END FOR

RETURN R
END
```

Supplementary Data 2. ToppGene's parameters.

Reference	PMID: 19465376
available at	https://toppgene.cchmc.org/prioritization.jsp.
Training Gene Set	list of differently expressed proteins
Test set	proteins of the string data base with At least 2 relations (12396)
pvalue method	probabibility density function
Feature	All, GO MF, GO BP, GO CP, Human phenotype, mouse phenotype, Pathway, pubmed, disease
Random sampling size	5000
min. feature count	2

Supplementary Data 3. Phenolyzer's parameters.

Reference	PMID: 26192085
available at	http://phenolyzer.wglab.org/
Disease/Phenotype:	autosomal dominant polycystic kidney disease type 1 OR ureteropelvic junction obstruction
gene selection	list of differently expressed proteins
region selection	no
phenotype interpret	phenotype interpretation
weight adjust	no
world cloud	no
Addon seed gene	disgenet disease gene mapping and genetic association data base
Addon gene relation	nothing selected
addon gene scores	gene haploinsufficiency score and gene intolerance score

Supplementary Data 4. Endeavour's parameters.

**Reference**	PMID: 27131783
available at	https://endeavour.esat.kuleuven.be/Endeavour.aspx
species	homo sapiens
training genes	list of differently expressed proteins
all data source	Gene and protein function , chemical information, biomolecular pathways, phenotypic information, interaction networks, expression profiles, expression ontologie, sequence based features
candidates	proteins of the string data base with at least 2 relations (12396)

Supplementary Data 5. MaxLink's parameters.

**Reference**	PMID: 24849579
available at	https://funcoup5.scilifelab.se/maxlink/maxlink.action
Gene identifier(s)	list of differently expressed proteins
Species	Homo sapiens
Confidence threshold	0.8
Candidates	200

Supplementary Data 6. ToppNet's parameters.

Reference	PMID: 19245720
available at	https://toppgene.cchmc.org/index.jsp
Training Gene Set	list of differently expressed proteins
Test set	proteins of the string data base with at least 2 relations (12396)
prioritization method	k-step markov
step size	6
training gene neighborhood distance	1

Supplementary references

1 Bakun, M. *et al.* Urine proteome of autosomal dominant polycystic kidney disease patients. *Clin Proteomics* **9**, 13, doi:10.1186/1559-0275-9-13 (2012).
2 Rauniyar, N. *et al.* Quantification of Urinary Protein Biomarkers of Autosomal Dominant Polycystic Kidney Disease by Parallel Reaction Monitoring. *Proteomics Clin Appl* **12**, e1700157, doi:10.1002/prca.201700157 (2018).
3 Lacroix, C. *et al.* Label-free quantitative urinary proteomics identifies the arginase pathway as a new player in congenital obstructive nephropathy. *Mol Cell Proteomics* **13**, 3421-3434, doi:10.1074/mcp.M114.040121 (2014).
4 Chen, H. *et al.* Quantitative Urinary Proteome Reveals Potential Biomarkers for Ureteropelvic Junction Obstruction. *Proteomics Clin Appl*, e1800101, doi:10.1002/prca.201800101 (2018).
5 Boca, M. *et al.* Polycystin-1 induces resistance to apoptosis through the phosphatidylinositol 3-kinase/Akt signaling pathway. *J Am Soc Nephrol* **17**, 637-647, doi:10.1681/ASN.2005050534 (2006).
6 Ni, Y., Sinnett-Smith, J., Young, S. H. & Rozengurt, E. PKD1 mediates negative feedback of PI3K/Akt activation in response to G protein-coupled receptors. *PLoS One* **8**, e73149, doi:10.1371/journal.pone.0073149 (2013).
7 Kunnen, S. J. *et al.* Comparative transcriptomics of shear stress treated Pkd1(-/-) cells and pre-cystic kidneys reveals pathways involved in early polycystic kidney disease. *Biomed Pharmacother* **108**, 1123-1134, doi:10.1016/j.biopha.2018.07.178 (2018).
8 Xu, J. X. *et al.* Polycystin-1 and Galpha12 regulate the cleavage of E-cadherin in kidney epithelial cells. *Physiol Genomics* **47**, 24-32, doi:10.1152/physiolgenomics.00090.2014 (2015).
9 Li, Z. *et al.* E-Cadherin Facilitates Protein Kinase D1 Activation and Subcellular Localization. *J Cell Physiol* **231**, 2741-2748, doi:10.1002/jcp.25382 (2016).
10 Conduit, S. E. *et al.* beta-catenin ablation exacerbates polycystic kidney disease progression. *Hum Mol Genet* **28**, 230-244, doi:10.1093/hmg/ddy309 (2019).
11 Wuebken, A. & Schmidt-Ott, K. M. WNT/beta-catenin signaling in polycystic kidney disease. *Kidney Int* **80**, 135-138, doi:10.1038/ki.2011.87 (2011).
12 Han, C. L. *et al.* A multiplexed quantitative strategy for membrane proteomics: opportunities for mining therapeutic targets for autosomal dominant polycystic kidney disease. *Mol Cell Proteomics* **7**, 1983-1997, doi:10.1074/mcp.M800068-MCP200 (2008).
13 Khan, S. *et al.* A role for PKD1 in insulin secretion downstream of P2Y1 receptor activation in mouse and human islets. *Physiol Rep* **7**, e14250, doi:10.14814/phy2.14250 (2019).
14 Vareesangthip, K., Tong, P., Wilkinson, R. & Thomas, T. H. Insulin resistance in adult polycystic kidney disease. *Kidney Int* **52**, 503-508, doi:10.1038/ki.1997.360 (1997).
15 Arnould, T. *et al.* The polycystic kidney disease 1 gene product mediates protein kinase C alpha-dependent and c-Jun N-terminal kinase-dependent activation of the transcription factor AP-1. *J Biol Chem* **273**, 6013-6018, doi:10.1074/jbc.273.11.6013 (1998).
16 Arnould, T. *et al.* Cellular activation triggered by the autosomal dominant polycystic kidney disease gene product PKD2. *Mol Cell Biol* **19**, 3423-3434, doi:10.1128/mcb.19.5.3423 (1999).
17 Le, N. H. *et al.* Increased activity of activator protein-1 transcription factor components ATF2, c-Jun, and c-Fos in human and mouse autosomal dominant polycystic kidney disease. *J Am Soc Nephrol* **16**, 2724-2731, doi:10.1681/ASN.2004110913 (2005).
18 Plouffe, S. W., Hong, A. W. & Guan, K. L. Disease implications of the Hippo/YAP pathway. *Trends Mol Med* **21**, 212-222, doi:10.1016/j.molmed.2015.01.003 (2015).
19 Wong, J. S., Meliambro, K., Ray, J. & Campbell, K. N. Hippo signaling in the kidney: the good and the bad. *Am J Physiol Renal Physiol* **311**, F241-248, doi:10.1152/ajprenal.00500.2015 (2016).
20 Aguiari, G. *et al.* Polycystin-1 regulates amphiregulin expression through CREB and AP1 signalling: implications in ADPKD cell proliferation. *J Mol Med (Berl)* **90**, 1267-1282, doi:10.1007/s00109-012-0902-3 (2012).
21 Idowu, J. *et al.* Aberrant Regulation of Notch3 Signaling Pathway in Polycystic Kidney Disease. *Sci Rep* **8**, 3340, doi:10.1038/s41598-018-21132-3 (2018).
22 Surendran, K., Selassie, M., Liapis, H., Krigman, H. & Kopan, R. Reduced Notch signaling leads to renal cysts and papillary microadenomas. *J Am Soc Nephrol* **21**, 819-832, doi:10.1681/ASN.2009090925 (2010).
23 Jeon, J. O., Yoo, K. H. & Park, J. H. Expression of the Pkd1 gene is momentously regulated by Sp1. *Nephron Exp Nephrol* **107**, e57-64, doi:10.1159/000108643 (2007).
24 Wang, Q. *et al.* Characterization of the polycystic kidney disease 2 gene promoter. *Genomics* **104**, 512-519, doi:10.1016/j.ygeno.2014.08.006 (2014).

25 Harris, P. C. & Torres, V. E. Genetic mechanisms and signaling pathways in autosomal dominant polycystic kidney disease. *J Clin Invest* **124**, 2315-2324, doi:10.1172/JCI72272 (2014).
26 Sweeney, W. E., Jr., von Vigier, R. O., Frost, P. & Avner, E. D. Src inhibition ameliorates polycystic kidney disease. *J Am Soc Nephrol* **19**, 1331-1341, doi:10.1681/ASN.2007060665 (2008).
27 van Bodegom, D., Roessingh, W., Pridjian, A. & El Dahr, S. S. Mechanisms of p53-mediated repression of the human polycystic kidney disease-1 promoter. *Biochim Biophys Acta* **1799**, 502-509, doi:10.1016/j.bbagrm.2010.04.001 (2010).
28 Van Bodegom, D. *et al.* The polycystic kidney disease-1 gene is a target for p53-mediated transcriptional repression. *J Biol Chem* **281**, 31234-31244, doi:10.1074/jbc.M606510200 (2006).
29 Li, H. *et al.* p53 mutation regulates PKD genes and results in co-occurrence of PKD and tumorigenesis. *Cancer Biol Med* **16**, 79-102, doi:10.20892/j.issn.2095-3941.2018.0170 (2019).
30 Hanatani, S. *et al.* Akt1-mediated fast/glycolytic skeletal muscle growth attenuates renal damage in experimental kidney disease. *J Am Soc Nephrol* **25**, 2800-2811, doi:10.1681/ASN.2013091025 (2014).
31 Rodriguez-Pena, A. B. *et al.* Activation of Erk1/2 and Akt following unilateral ureteral obstruction. *Kidney Int* **74**, 196-209, doi:10.1038/ki.2008.160 (2008).
32 Docherty, N. G. *et al.* Increased E-cadherin expression in the ligated kidney following unilateral ureteric obstruction. *Kidney Int* **75**, 205-213, doi:10.1038/ki.2008.482 (2009).
33 Yan, Y. *et al.* Src inhibition blocks renal interstitial fibroblast activation and ameliorates renal fibrosis. *Kidney Int* **89**, 68-81, doi:10.1038/ki.2015.293 (2016).
34 Liu, N. *et al.* Blocking the class I histone deacetylase ameliorates renal fibrosis and inhibits renal fibroblast activation via modulating TGF-beta and EGFR signaling. *PLoS One* **8**, e54001, doi:10.1371/journal.pone.0054001 (2013).
35 Noh, H. *et al.* Heat shock protein 90 inhibitor attenuates renal fibrosis through degradation of transforming growth factor-beta type II receptor. *Lab Invest* **92**, 1583-1596, doi:10.1038/labinvest.2012.127 (2012).
36 Dessapt, C. *et al.* Mechanical forces and TGFbeta1 reduce podocyte adhesion through alpha3beta1 integrin downregulation. *Nephrol Dial Transplant* **24**, 2645-2655, doi:10.1093/ndt/gfp204 (2009).
37 Wu, W. *et al.* Beta1-integrin is required for kidney collecting duct morphogenesis and maintenance of renal function. *Am J Physiol Renal Physiol* **297**, F210-217, doi:10.1152/ajprenal.90260.2008 (2009).
38 Arvaniti, E. *et al.* Whole-transcriptome analysis of UUO mouse model of renal fibrosis reveals new molecular players in kidney diseases. *Sci Rep* **6**, 26235, doi:10.1038/srep26235 (2016).
39 Silverstein, D. M. *et al.* Altered expression of immune modulator and structural genes in neonatal unilateral ureteral obstruction. *Kidney Int* **64**, 25-35, doi:10.1046/j.1523-1755.2003.00067.x (2003).
40 Lee, J. *et al.* p38 MAPK activity is associated with the histological degree of interstitial fibrosis in IgA nephropathy patients. *PLoS One* **14**, e0213981, doi:10.1371/journal.pone.0213981 (2019).
41 Stambe, C., Nikolic-Paterson, D. J., Hill, P. A., Dowling, J. & Atkins, R. C. p38 Mitogen-activated protein kinase activation and cell localization in human glomerulonephritis: correlation with renal injury. *J Am Soc Nephrol* **15**, 326-336, doi:10.1097/01.asn.0000108520.63445.e0 (2004).
42 Koca, O. *et al.* Analysis of expression of TNF-alpha and TGF-beta3 in intrinsic ureteropelvic junction obstruction. *Bratisl Lek Listy* **114**, 498-502, doi:10.4149/bll_2013_104 (2013).
43 Misseri, R. *et al.* Unilateral ureteral obstruction induces renal tubular cell production of tumor necrosis factor-alpha independent of inflammatory cell infiltration. *J Urol* **172**, 1595-1599; discussion 1599, doi:10.1097/01.ju.0000138902.57626.70 (2004).
44 Misseri, R. *et al.* TNF-alpha mediates obstruction-induced renal tubular cell apoptosis and proapoptotic signaling. *Am J Physiol Renal Physiol* **288**, F406-411, doi:10.1152/ajprenal.00099.2004 (2005).
45 Valles, P. G., Pascual, L., Manucha, W., Carrizo, L. & Ruttler, M. Role of endogenous nitric oxide in unilateral ureteropelvic junction obstruction in children. *Kidney Int* **63**, 1104-1115, doi:10.1046/j.1523-1755.2003.00833.x (2003).
46 Higgins, S. P. *et al.* TGF-beta1/p53 signaling in renal fibrogenesis. *Cell Signal* **43**, 1-10, doi:10.1016/j.cellsig.2017.11.005 (2018).
47 Yang, R. *et al.* p53 induces miR199a-3p to suppress SOCS7 for STAT3 activation and renal fibrosis in UUO. *Sci Rep* **7**, 43409, doi:10.1038/srep43409 (2017).
